# Development of Single Nucleotide Polymorphism (SNP)-Based Triplex PCR Marker for Serotype-specific *Escherichia coli* Detection

**DOI:** 10.3390/pathogens11020115

**Published:** 2022-01-19

**Authors:** Md-Mafizur Rahman, Sang-Jin Lim, Yung-Chul Park

**Affiliations:** 1 Division of Forest Science, Kangwon National University, Chuncheon 24341, Korea; mmrahman@btge.iu.ac.bd; 2 Faculty of Biological Science, Department of Biotechnology and Genetic Engineering, Islamic University, Kushtia 7003, Bangladesh; 3 Institute of Forest Science, Kangwon National University, Chuncheon 24341, Korea; sangjin@kangwon.ac.kr

**Keywords:** single nucleotide polymorphisms (SNPs), *Escherichia coli*, triplex primer, allele, genes, surveillance

## Abstract

Single-nucleotide polymorphisms (SNPs) are one of the most common forms of genetic variation and as such are powerful tools for the identification of bacterial strains, their genetic diversity, phylogenetic analysis, and outbreak surveillance. In this study, we used 15 sets of SNP-containing primers to amplify and sequence the target *Escherichia coli*. Based on the combination of the 15-sequence primer sets, each SNP site encompassing forward and reverse primer sequences (620–919 bp) were aligned and an SNP-based marker was designed. Each SNP marker exists in at least two SNP sites at the 3′ end of each primer; one natural and the other artificially created by transition or transversion mutation. Thus, 12 sets of SNP primers (225–488 bp) were developed for validation by amplifying the target *E. coli*. Finally, a temperature gradient triplex PCR kit was designed to detect target *E. coli* strains. The selected primers were amplified in three genes (*ileS*, *thrB,* and *polB*), with fragment sizes of 401, 337, and 232 bp for *E. coli* O157:H7, *E. coli*, and *E. coli* O145:H28, respectively. This allele-specific SNP-based triplex primer assay provides serotype-specific detection of *E. coli* strains in one reaction tube. The developed marker would be used to diagnose, investigate, and control food-borne *E. coli* outbreaks.

## 1. Introduction

Single-nucleotide polymorphisms (SNPs) are single-base differences in DNA between individual organisms [1,2,3,4]. They enable the distinction of very closely related organisms or very limited allelic differences on similar genomic structures, such as different serotypes of *Escherichia coli* bacteria [5,6]. SNPs are one of the most useful molecular markers because of their stability and abundance in all organisms. The advent of whole-genome sequencing (WGS) technologies [7,8], wider open-source websites, i.e., PubMLST [9], and the availability of reference genome sequences of many bacteria has allowed for the wider implementation of SNP-based detection [10,11,12]. Nucleotide substitutions during DNA replication of bacteria, with mutation rates of approximately 10^−9^ changes per nucleotide per generation, are important biologically informative markers in bacteria [13,14]. In addition, SNP data have also been used in epidemiological studies of field outbreaks [15]. However, the differentiation of bacteria at the serovar level remains challenging, and SNP-based approaches for differentiating specific strains have been applied to various bacterial species, such as *Escherichia* [16,17], *Salmonella* [18], *Brucella* [19,20], and *Bacillus* species [21]. To date, several molecular methods have been used for the detection of *E. coli* [3,10,11,17,22,23]. However, SNP-based techniques have become increasingly attractive for the efficient detection of *E. coli* compared to other recognized molecular techniques [13]. Using differences in SNPs of genes between isolates is also a promising technique for the identification of geographical origins of *E. coli* [13,24,25,26]. SNPs are abundant in microbes and are often considered the optimum choice for genetic studies, having several advantages such as flexibility, reduced error rate, speed, and cost-effectiveness [27]. SNPs within different *E. coli* genes may be useful markers for the development of rapid surveillance of food-borne diseases, outbreak tracing [26,28] and typing methods, routine diagnostics, and the typing of *Salmonella* [29]. This approach has already been useful in retrospective investigations [27,28,30,31].

Shiga toxin-producing *E. coli* (STEC), *E. coli* O157 has been a major food-borne pathogen since the early 1980s; however, early and accurate diagnosis presents preventive measures to minimize the risk of food-borne pathogens and their outbreaks [32,33]. *E. coli* O157:H7 has been reported in several outbreaks worldwide, in both developing and developed countries, including in the USA [34], Canada [35], and Europe [36]. Currently, there several molecular techniques are commonly used for food-borne disease surveillance and the subtyping of *E. coli*, such as the PulseNet [37,38], Multilocus Sequence Typing [39], and Multilocus Variable-Number Tandem-Repeat Analysis [40] techniques. PulseNet is based on the characterization of whole bacterial genomes using enzymatic digestion patterns that are separated by pulsed-field gel electrophoresis [12,20,38]. This technique can be biased because of the subjective interpretation of band patterns [37,41]. Similarly, Multilocus Sequence Typing (MLST) is widely used as a genotyping method and has been applied successfully to *E. coli* [22,23]; however, this sequencing procedure is expensive and time-consuming for routine monitoring [42]. Multilocus Variable-Number Tandem-Repeat analysis is another subtyping technique used for *E. coli*, but it has limitations in the development of a universal panel for MLVA [40]. Nevertheless, SNP analysis enables the classification of very similar genomes and the detailed classification of genomes of *E. coli* that are difficult to distinguish using conventional typing techniques [43]. To overcome the limitations of the abovementioned molecular methods, SNP-based molecular methods have been developed. Therefore, by comparing it with the existing methods, the SNP-based method can be used to develop accurate, rapid, and molecularly meaningful typing methods that may lead to the more efficient detection of serotype-specific food-borne pathogenic *E. coli*. In this study, we altered a mismatched nucleotide within the three bases closest to the 3′ end (SNP site) in each primer sequence to detect serotype-specific *E. coli*. The aim of this study was to investigate the interrogation of informative SNPs in genes of WGS sequences of *E. coli*, along with the development of SNP marker-based detection.

## 2. Results

### 2.1. Acquisition and Alignment of WGS of E. coli from GenBank

A total of six *E. coli* strains, including three O157 and non-O157 sequences, was downloaded from GenBank. From these, *E. coli* str. K-12 contained the most known genome sequences and was therefore selected as the reference strain, whereas the remaining five *E. coli* strains were considered for comparison. The accession numbers of all six *E. coli* strains are provided in Table S1.

### 2.2. Search of SNP Sites from NGS of E. coli Genomes in GenBank

The six *E.* *coli* genome sequences were downloaded and compared to the reference genome sequence. A total of 2160 SNPs were identified from the alignment of *E. coli* WGS (Table S1). From them, the high-quality, useful, and abundant SNP sites were selected based on nonsynonymous mutation with bioinformatics software, and 15 sets of SNP sites encompassing forward and reverse primers were thus selected. These 15 primer sets were chosen from 11 different genes of the *E. coli* genome (*thrB/C, nhaR, ileS, dapB, carB, caiB/C, polB, araB, yabI, thiP*, and *leuD*) (Table 1). The ambiguous codes and positions of SNPs with a nonsynonymous amino acid of a specific gene and the reference genome of non-O157 *E. coli* (NC_000913) are shown in Table 1.

### 2.3. Amplification of SNP Sites and Sequencing of Target E. coli Strains Using Newly Designed Primers

**Figure 1:**
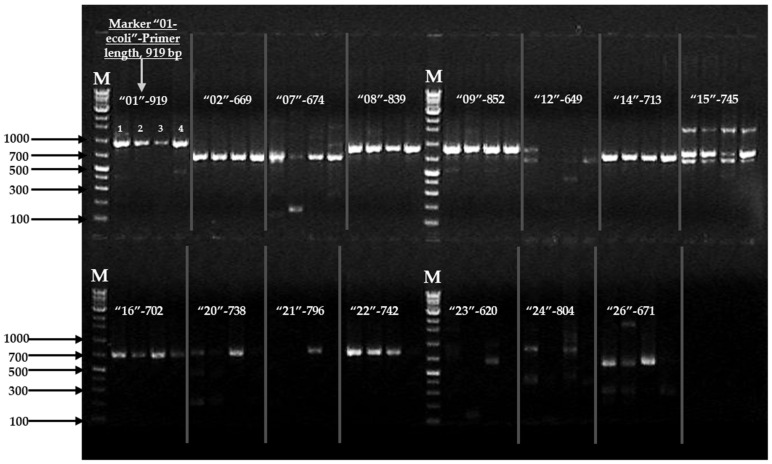
PCR amplification of three different serotypes including four target *Escherichia coli* strains with 15 primer sets of first PCR amplification {(01-ecoli-F/R (919 bp); 02-(669 bp); 07-(674 bp); 08-(839 bp); 09-(852 bp); 12-(649 bp); 14-(713 bp); 15-(745 bp); 16-(702 bp); 20-(738 bp); 21-(796 bp); 22-(742 bp); 23-(620 bp); 24-(804 bp) and 26-(671 bp)}. PCR band ‘M’ indicates DNA 100 bp marker. The gel lane numbers are shown in each section (1–4): lane No. 1 = *E. coli* O157:H7 (ATTC-95150); No. 2 = *E. coli* O157:H7 (NCCP-15739); No. 3 = *E. coli* (KVCC-BA1800069); No. 4 = *E. coli* O145:H28 (KVCC-BA1800090). The four primers (12-, 21-, 23-, and 24-ecoli primer sets) were not produced with any desired bands with the target genes of *E. coli*, whereas the three primers (14-, 15- and 26-ecoli primer sets) were not produced with the target genes of *E. coli*. The detailed information of all primers was provided in Table 1, Tables S2 and S3.

A total of 15 SNP markers with a size of 620–919 bp were identified from *E. coli* genomes via the bioinformatics software analysis. Within the SNP markers, SNP sites, bold ambiguous codes, and the positioning of the amino acid codes in each gene of the reference *E. coli* genome and with the respective genes are marked in Table 1. All 15 primers were tested for the amplification of the SNP markers with the target *E. coli* strains (Table 1). The amplified product sizes and an image of the PCR results are shown in Figure 1.

### 2.4. Confirmation of SNP Sites Based on Aligned Sequences of Target E. coli and Design of E. coli Serotype-Specific SNP Primers Using Sequences Containing SNP Sites

During the first PCR amplification, seven primer sets (01-, 02-, 07, 08-, 09-, 16-, and 22-ecoli) were amplified on the target genes of the tested *E. coli* strains (Tables S2 and S3), whereas four primer sets (12-, 21-, 23-, 24-ecoli) were not amplified on any target bands (Figure 1) and three primer sets (14-, 15-, and 26-ecoli) were partially amplified on the target genes of four *E. coli* strains (Figure 1, Tables S1 and S3). Moreover, the primers of 20- and 22-ecoli were amplified with all the tested *E. coli* strains during the second PCR, and their sequences are shown in Table S2. Thus, we tested all primers with all target *E. coli* strains with the first and second PCR amplification and each primer was amplified the specific gene (i.e., 01-primer amplified specific gene, homoserine kinase, *thrB*) of the four target *E. coli* strains.

The amplified products of all 15 primers, their sequences, and the detailed information of each primer with the four target *E. coli* strains are shown in Tables S2 and S3. The specific gene target amplified sequences (*n =* 38 out of 60) were submitted to the NCBI database with the following accession numbers (OL589326–OL589363). The chimeric, off-target sequences with non-specific genes and dimer sequences (*n =* 22) were not used for further analysis of the SNP-based primer design (Table S4). Moreover, the three positions of SNPs were 22,945 > C, 23,104 > A, and 23,137 > A of *ileS* gene in reference non-O157 *E. coli* str. K-12 (NC_000913) (Table 1 and Table S1). The amplified *ileS* gene products of the target *E. coli* strains were sequenced and aligned. We then checked one or multiple SNP positions on the aligned sequences. Thus, 12 sets of SNP primers of serotype-specific *E. coli* were designed based on five genes (*nhaR*, *thrB/C ileS caiB*, and *polB*) where at least one natural SNP was present (Table 2).

### 2.5. E. coli-Specific SNP Primer Design Using Aligned Gene Sequences with Availability of SNP Sites

**Figure 2:**
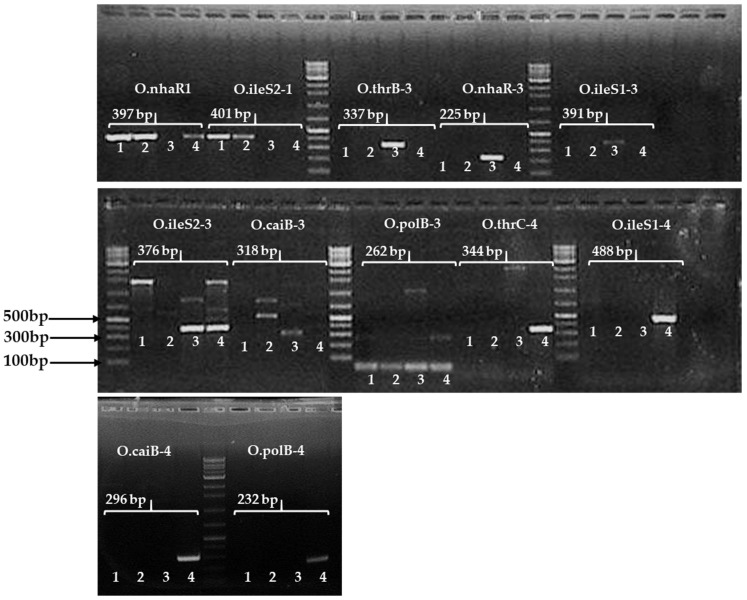
PCR amplification of SNP-based markers with four target *E. coli* strains including two *E. coli* O157, *E. coli*, and *E. coli* O145. PCR band ‘M’ indicates DNA of 100 bp. The gel lane numbers are provided in each section (1–4): lane No. 1 = *E. coli* O157:H7 (ATTC-95150); No. 2 = *E. coli* O157:H7 (NCCP-15739); No. 3 = *E. coli* (KVCC-BA1800069); No. 4 = *E. coli* O145:H28 (KVCC-BA1800090). The nine primers (O.nhaR1, O.ileS2-1, O.thrB-3, O.nhaR-3, O.ileS1-3, O.thrC-4, O.ileS1-4, O.caiB-4, and O.polB-4) produced a single target band, whereas three primers (ileS2-3, O.caiB-3, O.polB-3) produced dimers or multiple bands. The two primers (O.nhaR1 and O.ileS2-1) produced the target band of serotype specific *E. coli* O157:H7 but O.nhaR1 also produced the target band of *E. coli*. Moreover, the primers (ileS1-3, O.caiB-3, O.polB-3, and O.polB-4) produced a light band during the 1st PCR and these three primers produced a strong band with the 2nd PCR amplification (images are not shown in here but 1st and 2nd PCR amplification sequences are provided in Tables S2 and S4). Moreover, the detailed primer information is provided in Table 2.

Based on previous research [44], the triplet base of the 3′ end primer of each primer sequence and the last triplet codon at the 3′ end of the first and third position base altered within all primer sets so that they could hybridize very strongly during PCR amplification. Based on this principle, we designed 12 sets of SNP primers (225–488 bp) developed for validation by amplifying target *E. coli* strains. Two examples of these primers are shown in Figures S1 and S2. The marker “01”-amplified homoserine kinase (*thrB*) genes of four *E. coli* strains were aligned. It was found that the number of the natural SNP position was 342 and the artificial transition (purine to purine)-mutated SNP position number was 340 on the aligned gene sequences. Thus, we designed the SNP based marker ‘O-thrB-3-F/R’ to include at least two SNPs, e.g., the forward primer length was 18 bp from 325 to 342 (the position of the natural SNP ‘G = 342′ and the transition-altered SNP site was “G = 340; A > G”) and the reverse primer length was 20 bp from 642 to 661 (the position of two natural SNPs ‘T = 642′ and ‘C = 643’, respectively, and the transition-mutated SNP site was “G = 645; A > G”). The amplified target product size was 337 bp (Figure S1). Another forward primer ‘ileS1-4-F’ was 19 bp long (141–159) and the reverse primer ‘ileS1-4-R’ was 20 bp long (609–628), as shown in Figure S2. The target amplicon size was 488 bp. Moreover, the amplicon size (391 bp) and the position of the forward and reverse primers of the primer “ileS1-3” are shown in Figure S2. The natural SNP is marked by a square shape and the altered SNP is marked by a black shaded italicized square shape (Figures S1 and S2). Thus, 12 SNP-based markers of *E. coli* serotype-specific primers were used for amplification with the four *E. coli* strains, and finally, it was possible to confirm selection with the efficiency of the SNP marker of the positive target band of all *E. coli* strain-specific SNP primers for PCR amplification (Table 3 and Figure 2).

### 2.6. SNP Marker-Based Triplex PCR

**Figure 3:**
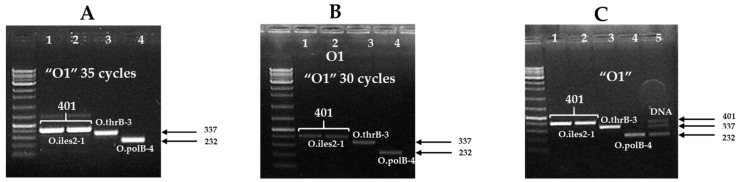
PCR amplification of serotype-specific triplex *E. coli* primer set (O1). The gel lane numbers are shown in each section (1–4): No. 1 = *E. coli* O157:H7 (ATTC-95150); No. 2 = *E. coli* O157:H7 (NCCP-15739); No. 3 = *E. coli* (KVCC-BA1800069); No. 4 = *E. coli* O145:H28 (KVCC-BA1800090), and No. 5 = Genomic DNA without PCR of *E. coli*. (**A**) A strong band was produced by 35 PCR cycles with the serotype-specific *E. coli*; (**B**) a weak band was produced by 30 PCR cycles with the serotype-specific *E. coli*; (**C**) band with isolated genomic DNA of three serotype-specific *E. coli* without PCR amplification. In each lane with a band, the image was produced by using the concentration of 5 μL PCR products.

To determine the strain specificity of the confirmed SNPs, we aligned the re-sequences containing the variable positions of the target *E. coli* sequences (Table S1). To detect the target serotypes of *E. coli*-specific SNP markers in a single reaction, a temperature gradient SNP marker-based triplex PCR kit was developed. This efficient test was performed with target *E. coli* strains via PCR amplification of the SNP-based triplex PCR kit (Figure 3 and Table 4).

In the encompassing primer sequences (Table 1), the red-colored bases indicate natural SNPs and the blue-colored bases indicate artificial SNPs (altered by transition or transversion mutation) (Table 2). For example, for the primer of O.ileS2-1F “GATCATCTTCCGCGC**AG**C**G**”, which consists of three SNP bases, the underlined nucleotide positions are the 16th, ‘A’, and the 19th, ‘G’, whereas the blue color base, the 17th, was changed from ‘A’ to ‘G’ (transition). Thus, in each primer sequence of the SNP sites, we altered an artificial base, except for O-polB-4-F, so that SNP-based primers improved their ability to bind the PCR template sequence, thereby improving the allele-specific detection of target *E. coli* strains (Table 2, Figure 2). The 12 sets of SNP-based primers were designed to amplify the desired band of serotype-specific *E. coli*. Finally, SNPs containing three genes (*ileS*, *thrB,* and *polB*) with three markers (O.ileS2-1, O.thrB-3, and O.polB-4) were selected to detect three target *E. coli* strains (Figure 3 and Table 4). In the case of *E. coli* O157:H7, two SNP-based primers (O.nhaR1 and O.ileS2-1) produced the target band, whereas the other two markers (O.ileS2-3 and O.caiB-3) produced the off-target bands (Table 3). The primer ‘O.ileS2-1′ detected two *E. coli* O157:H7 strains, but it did not detect the other two non-O157 *E. coli* strains. Thus, this marker (O.ileS2-1) is a good candidate for *E. coli* O157:H7. However, if we want to know the serotypes of an unknown *E. coli*, we have to PCR-amplify each of 12 primer pairs, which is a limitation of the developed SNP-based (*n* = 12) marker. We overcame the cost and time involved in repeated PCR amplification of the SNP-based marker through the development of a triplex SNP marker (the developed triplex PCR marker is indicated as “O1”) (Figure 3 and Table 4).

Serotype-specific *E. coli* strains were detected in a single reaction with the developed assay (Figure 3 and Table 4). In addition, the marker ileS2-1 was able to detect the most pathogenic O157, whereas the other two primer pairs, O.thrB-3 and O.polB-4, could detect *E. coli* (KVCC-BA1800069) and *E. coli* O145:H28 (KVCC-BA1800090), respectively (Table 4).

### 2.7. Cross Reaction and Validation Test

The developed markers were investigated by means of the cross-reaction test with other gram-positive and gram-negative pathogenic bacteria. The developed assay did not produce cross-reactions with any of the tested bacteria, indicating the specificity of the primer sets. However, the assay sometimes produced an off-target band with the tested bacterial strains (data not shown). For more validation, 23 identified *E. coli* from wild-animal fecal samples were tested and validated with SNP-based markers. The generating band was marked on the isolate lanes numbered from one to eight, where lane 1 is a positive band of reference *E. coli* O157 (401 bp) and the tested *E. coli* (lane no. 2–7) was matched with *E. coli* O157:H7. Lanes no. 9–11 were matched to the target *E. coli* (KVCC-BA1800069, lane no. 13, amplicon length 337 bp). Lanes no. 14–19 were matched to the target *E. coli* O145:H28 (KVCC-BA1800090; lane no. 14, amplicon length 232 bp). Five of the isolates (lanes no. 20–25) were not exactly matched to the three target *E. coli* strains. They might have originated from the animal fecal samples as different *E. coli* strains (Figure S3). Sometimes, the tested *E. coli* was produced multiple off-target bands. To obtain a clearer resolution, we should conduct further analysis with a variety of *E. coli* isolates with diverse sources, such as foods.

## 3. Discussion

To date, several different methodological approaches have been used to detect *E. coli*, for instance, PCR bands [45], PFGE [46], phage typing [47], and MLST [18,23]. There are some limitations to the current molecular typing methods; however, SNP-based techniques have recently been suggested as a cost-effective alternative typing method for various bacterial species, as well as *E. coli* [6,27,28,48]. The SNPs in the WGS have discriminative power that enables the comparison of genetic bases not only between bacterial subspecies but also at the serotype level. This provides an easy method of determining the position of SNPs from the WGS of bacteria using different bioinformatics software tools. Thus, various polymorphisms can be used to detect very similar strains from different sources [2,3]. In addition, SNPs could be used as an accurate and convenient method to detect disease outbreaks, for the surveillance of food-borne pathogens [13,26] and their source detection [28,34], to develop risk models for outbreaks, and even to map the phylogenetic and evolutionary relationships between similar strains [49].

In this study, fifteen primer sets were chosen from 11 different genes of the *E. coli* genome (Table 1). Primers were designed based on suitable abundant SNP sites on the aligned WGS of the 11 abovementioned genes, approximately 620–919 bp (Table 1). Grish and Burbudae [50] conducted a similar study and found that nine markers were considered to be the best candidate markers in terms of their target band patterns out of the 30 SNP markers tested. In this study, SNP-based primers were designed based on natural and artificial SNPs or by introducing a mismatched nucleotide within the three bases at the 3′ end SNP sites (transition or transversion mutations). In addition, the artificial mismatched (transition or transversion) bases that were varied in the 3rd position of a codon at the 3′ end of each primer might have altered the codon, resulting in an amino acid substitution, which represents a target for PCR methods [51,52,53]. Moreover, the introduction of a mismatched (A-T transversion or A-G transition) base pair at the third base from the 3′ end could increase the allele-specific amplification during PCR [44,54,55]. Thus, transitions (A-G and T-C), as well as transversions (A-T, A-C, G-C, and G-T), were useful as base pair mismatches in improving the allele-specific amplification. Based on natural and artificial bases (transition/transversion), we developed 12 sets of SNP primers (232–488 bp) for validation by amplifying the four target *E. coli* genes including three different serotype strains (Table 2). Moreover, the melting temperature (Tm) sometimes depends on the GC content of the designed primer sequences, which is important for the adjustment of PCR conditions. By introducing transverse mismatched bases, the melting temperature of allele-specific SNP-based primers can be fixed or adjusted to standardized PCR conditions [44]. Similarly, the *E. coli* O157 and non-O157 detected primers were designed based on natural and artificial SNPs or a mismatched nucleotide introduced within the three bases (except for primer ‘O.polB-4-F’) at the 3′ end SNP sites (Table 2, Figure 2). A temperature gradient SNP marker-based triplex PCR kit was developed for the target serotypes of *E. coli* specific SNP markers in a single reaction. An efficient test was performed with the target *E. coli* serovar by means of PCR amplification of the SNP-triplex PCR kit and was adjusted to standardized PCR conditions (Table S5). In addition, the *E. coli* detected in wild-animal fecal samples were tested with the efficiency and validation of the designed primer (O1), but all the isolates were not exactly matched to the three target *E. coli* serotype strains (Figure S3). We should analyze more isolates for the validation of the SNP-based triplex O1 primer.

Recently, software algorithms and parameters have been used to search for SNP positioning from raw or assembled genome sequences [56,57]. SNP-based techniques have become increasingly attractive for the efficient detection of *E. coli*, compared to other molecular techniques. In some previous investigations, the SNP-based technique has already been useful in retrospective research studies based on SNPs of WGS that can detect different isolates of similar bacterial strains [2,30,31,58]. In one study, two highly homologous serovars were distinguished based on 20 SNPs compared to the sequence of a reference strain [59]. Nonetheless, another study showed that up to four SNPs were required for the precise identification of all *E. coli*-investigated serovars. Dallman et al. showed three SNP differences in outbreak-associated isolates compared to non-outbreak isolates. It was possible to detect outbreak isolates using SNP-based primers [60]. Current SNP-based typing methods can be applied for the detection and typing of STEC and other organisms. [24]. Desphande et al. showed that 29 SNPs were used as a signature sequence (either synonymous or non-synonymous amino acid changes) for *E. coli* strains from other pathogenic serotyping *E. coli* strains present in the NCBI database [61]. In our research, we mention the varying nucleotide positions (SNPs) between the following strains: IAI39, 2011C-3493, Sakai, NRG 857C, and UMN026, and the reference strain (K-12) (Table S1). A study by Camprubí-Font et al. compared 286 polymorphic sites in adherent-invasive *E. coli* (AIEC-associated SNPs). Sixty SNPs were selected for re-sequencing, and 20 of the confirmed SNPs in 11 genes of AIEC strains were used for the identification of *E. coli* [2]. Similarly, we found 2160 SNPs on the aligned genome of six serotype-specific *E. coli* strains, among which the SNP-flanking regions of aligned sequences on 11 genes were selected for further sequencing and finally only three SNPs containing three genes (*ileS2*, *polB*, and *thrB*) were confirmed for the SNP-based triplex PCR marker. However, Moorhead et al. speculated that PCR enzymes are capable of three to five proofreading activities to correct an artificial mismatch [51] but DNA polymerase can extend primers less efficiently (100- to 10,000-fold) compared to matched with mismatched 3′ bases [62]. The simultaneous introduction of mismatched bases in the third position prevented the amplification of fragments from the targeted *E. coli* genome (Table 2). Therefore, altered SNPs could be used for developing an alternative, accurate, rapid, and cost-effective typing method that may lead to significant improvements in the diagnosis of *E. coli*. To the best of our knowledge, this study is the first to detect serotype-specific *E. coli* strains using the developed (O1) SNP triplex PCR method.

## 4. Materials and Methods

### 4.1. General Overview of SNP-Based Marker Design and Validation with PCR Amplification of Target E. coli

**Figure 4:**
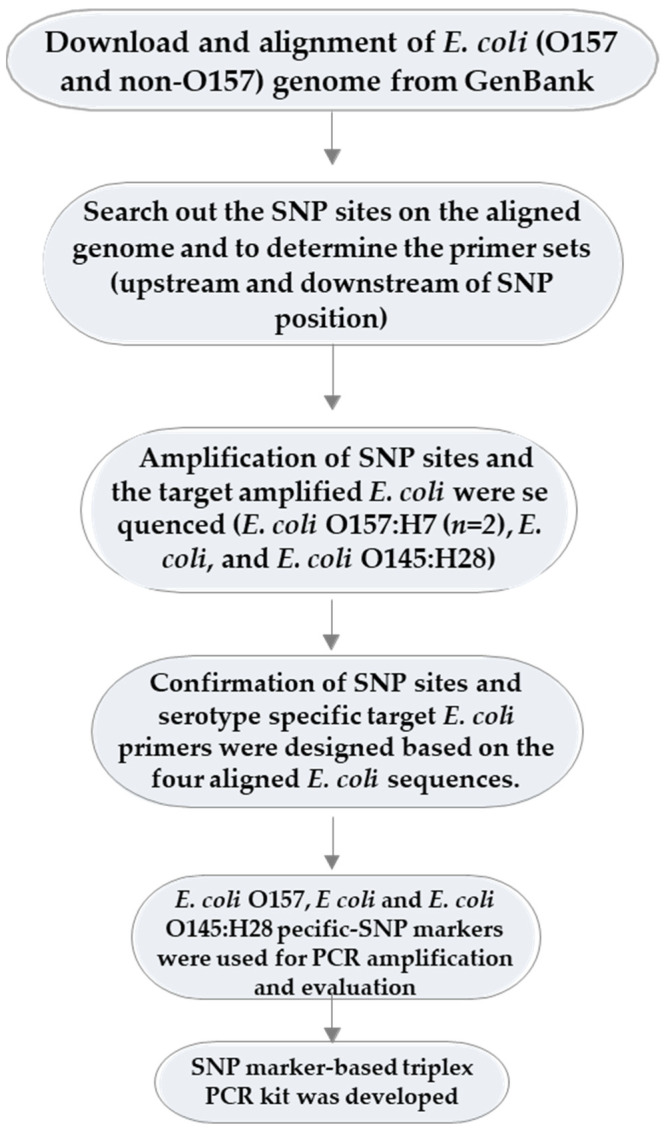
A schematic flow diagram of the development of the SNP-based marker for serotype-specific *E. coli* detection.

There were six steps involved in the development of SNP-based markers. They were as follows: (i) the whole-genome sequences of six *E. coli* strains, including three *E. coli* O157 and three non-O157 strains, were retrieved from the GenBank database and each was aligned with the respective reference (K-12 substr. MG1655). (ii) We used the NUCmer program [63] to search for the SNP positions on the aligned WGS and to determine the primer sets encompassing each SNP site (upstream and downstream of each SNP position). The SNP-matrix files were shown in variant call format (VCF) files by generating them from VCFtools v.4.1.0 (https://vcftools.github.io/index.html, accessed on 29 December 2021), and a filtered SNP matrix was constructed from the output VCF files [63]. (iii) The designed primers were used for the amplification of SNP sites, and subsequently, the four amplified *E. coli* were sequenced (*E. coli* O157 and non-O157). (iv) Then, *E. coli* primers were designed based on the four aligned *E. coli* sequences. (v) *E. coli* O157 and non-O157 specific-SNP markers were used for PCR amplification and evaluation. (vi) Finally, the SNP marker-based triplex PCR kit was designed. A schematic flow diagram of the development of the SNP-based marker for *E. coli* detection is shown in Figure 4.

### 4.2. Culture and Isolation of Genomic DNA from Serotype-Specific E. coli (O157 and Non-O157) Strains

The four *E. coli* strains, including three different serotype strains, were selected for DNA isolation, PCR amplification (with the designed SNP-encompassing primers), and sequencing. The two *E. coli* O157 strains were O157:H7 (ATCC-95150, NCCP-15739) and the non-O157 strains were *E. coli* (KVCC-BA1800069) and *E. coli* O145:H2 (KVCC-BA1800090). The target bacterial colonies were streaked onto nutrient agar media, and a single colony was collected using a sterilized toothpick. The colonies were incubated at 35 °C for 18 h in a 5 mL lactose broth (LB) solution. Genomic DNA was extracted from 1 mL of LB culture fluid using a DNeasy Blood and Tissue Kit, according to the manufacturer’s instructions (Qiagen, Valencia, CA, USA).

### 4.3. Acquisition and Alignment of WGS of E. coli from GenBank

The complete whole-genome sequences of *E. coli* based on NGS data were downloaded from GenBank (ftp://ftp.ncbi.nlm.nih.gov/genomes/refseq/, accessed on 10 July 2021), including one reference strain of the most studied and best-annotated genome (*E. coli* str. K-12), which was used as a reference genome. *E. coli* str. K-12 substr. MG1655 (NC-000913.3) was downloaded along with five query strains, *E. coli* IAI39 (NC_011750), *E. coli* O104:H4 str. 2011C-3493 (NC-018658), *E. coli* O157:H7 str. Sakai (NC-002695), *E. coli* O83:H1 str. NRG 857C (NC-017634), and *E. coli* UMN026 (NC-011751). Among them, three were *E. coli* O157 and three were non-O157. We selected the most suitable SNPs based on the allelic diversity of the aligned sequences with SNP sites encompassing a primer compared to the reference and query sequences of *E. coli* (Table S1). The “selected SNPs” are defined as the homologous gene sequences of aligned pairs of WGS. In addition, the SNPs in the highly variable region end of the contigs or synonymous amino acid changes in the coding regions were not selected.

### 4.4. Search for SNP Sites on WGS Alignment and Design with Suitable Primers Encompassing SNP Sites (Upstream and Downstream of SNP Sites)

We used the NUCmer software of MUMer v4.0.0 (https://mummer4.github.io/, accessed on 12 July 2021) to determine the SNP positions on WGS alignments and to design primers both upstream and downstream of SNP sites [42,63,64]. The construction of the indel matrix and the detection of SNPs were completed using VCFtools v.4.1.0 (https://vcftools.github.io/index.html, accessed on 15 July 2021), and variant annotation was performed using SnpEff v.4.3.0 (http://pcingola.github.io/SnpEff/, accessed on 17 July 2021). The final output of the workflow was a filtered SNP matrix. SNP positions were inferred using show-snp programs [63]. Eleven genes were selected based on the suitable SNP sites. These genes (homoserine kinase-*thrB*, threonine synthase-*thrC*, transcriptional activator protein-*nhaR,* isoleucine-tRNA ligase-*ileS*, 4-hydroxy-tetrahydrodipicolinate-*dapB*, carbamoyl-phosphate synthase-*carB*, DNA polymerase-*polB*, ribulokinase-*araB*, inner membrane protein-*yabI*, thiamine transport system permease protein-*thiP*, and 3-isopropylmalate dehydratase small subunit-*leuD*) were amplified with four *E. coli* strains, which were selected as targets for sequence analysis for the desired SNPs (Table 1). The gene sequences were selected based on non-synonymous changes in amino acids. The “selected SNPs” were validated by re-sequencing with Sanger sequencing. The primers were designed upstream and downstream of the selected SNP position to achieve good sequence quality containing SNPs, using Primer3 software v0.4.0 (http://bioinfo.ut.ee/primer3-0.4.0/, accessed on 19 July 2021). Multiple primer sets were produced but the suitable primer sets were selected based on corresponding SNP positions and amplicon length (approximately 750–1000 bp).

### 4.5. Amplification of DNA Fragments Encompassing the SNP Sites of Interest and Re-Sequencing of the Amplified PCR Products of the Target Strains Using the Newly Designed Primers

The target *E. coli* and laboratory-detected *E. coli* from wild-animal fecal samples were streaked onto nutrient agar media, and a single colony was then incubated at 35 °C for 18 h in a 5 mL lactose broth (LB) solution. Genomic DNA was extracted from 1 mL of LB culture fluid using a DNeasy Blood and Tissue Kit, according to the manufacturer’s instructions (QIAGEN Inc, Valencia, CA, USA). Then the target *E. coli* strains were selected for re-sequencing (Table S2) using newly designed primers for the amplification of the SNP sites that were aligned with six WGS GenBank datasets (https://www.ncbi.nlm.nih.gov/genome/?term=E.+coli, accessed on 10 July 2021). Information on the whole genome of *E. coli* is provided in Supplementary Table S1. Each PCR reaction consisted of 1 μL (5 ng/μL) of template DNA, 0.5 μL each of forward and reverse primers, 3 μL 10× HS buffer, 3 μL dNTP, 0.3 μL Hot Star *Taq* DNA polymerase (Qiagen), and 21.7 μL distilled water to a final volume 30 μL. The first and second cycles of PCR consisted of amplification at 95 °C for 5 min, followed by 30–35 cycles of denaturation for 30 s, annealing at 55 °C for the 1st PCR cycle and 50 °C for the 2nd PCR cycle with 30 s, polymerization at 72 °C for 1 min 30 s, and a final elongation at 72 °C for 10 min. The amplified product sequences are provided in Table S2 and Figure 1. The amplified primer products were purified (Gel & PCR Purification Kit; Biomedic Co., Ltd., Seoul, Korea) and sequenced using a BigDye Terminator v3.1 Cycle Sequencing Kit (Applied Biosystems, Foster City, CA, USA) and an ABI 3730 DNA Analyzer (Applied Biosystems, Foster City, CA, USA). All 11 genes, including SNP sites, were amplified using the target *E. coli*. The amplified PCR products of the target *E. coli* were re-sequenced (Table S2) and aligned using BioEdit Sequence Alignment Editor, version 7.0.0 (Tom Hall, North Carolina State University, Raleigh, NC, USA). However, none of the SNP-encompassing primers produced the desired SNPs of the expected bands or sequences due to the presence of short or chimeric sequences. The SNP sites on the aligned target *E. coli* of 11 gene sequences were confirmed. The *E. coli* detected primers were designed based on natural and altered SNPs or a mismatched nucleotide introduced within the three bases at the 3′ end SNP sites (transition or transversion mutations). Thus, all the designed primers were further analyzed using NetPrimer (https://www.premierbiosoft.com/netprimer/, accessed on 22 July 2021) to select the optimal primer pairs. The validated polymorphisms are referred to as “confirmed SNPs”.

### 4.6. PCR Amplification of E. coli O157 and Non-O157 Specific SNP Markers and Their Efficient Testing

Standard PCR primers targeting numerous SNP locations were initially designed through an in silico approach, and the primers were optimized via repeated PCR testing. To improve detection efficiency, we introduced a mismatched base within the triplet base of the 3′ end primer of each primer sequence. It was observed that there were no non-specific bands during PCR amplification [55]. Optimization was accomplished through repeated PCR cycling through variations in primer design, assay conditions, reagent concentrations, and the selection of alternative SNP targets. For this, the designed SNP primers were used to amplify the target *E. coli* strains. When SNPs were found with ambiguous or overlapping peaks, they were removed from further analysis. Finally, it was possible to confirm efficient SNP-based primers with the expected target bands of *E. coli* strains. Therefore, allele-specific primers were designed to discriminate single base changes through experimental optimization [54,55].

### 4.7. Development of a Triplex SNP-Based PCR Marker

For the detection of all target *E. coli* strain-specific SNP markers in a single reaction, a temperature gradient SNP-based triplex PCR kit was developed. Each PCR reaction consisted of 1 μL (5 ng/μL) of template DNA, 3 μL each of forward and reverse primers, DSbio Hot Start *Taq* mixture 10 μL, and 6 μL distill water to a final volume 20 μL. (Table S4). The PCR reaction was performed for a period of 5 min at 95 °C, followed by denaturation for 30–35 cycles for 30 s, then annealing at 55 °C for 35 s, polymerization at 72 °C for 1 min 30 s, and a final elongation at 72 °C for 10 min. The amplified product size and PCR results are shown in Figure 2 and Table S1.

### 4.8. Validation and Cross Reaction Test with SNP-Based Triplex PCR

Wild-animal fecal samples were collected from various agricultural regions across South Korea (unpublished data). From these fecal samples, *E. coli* isolates were detected based on cultural, serological, and molecular approaches as per the methods described previously [65]. For efficiency tests, the laboratory-isolated selective *E. coli* (*n* = 23) was tested with the triplex PCR marker (Figure S3). In addition, possible cross-reactions were also investigated with other closely related Gram-negative and Gram-positive bacteria (data not shown). The Gram-positive bacteria were *Staphylococcus aureus* (NCCP-14780); *Bacillus cereus* (NCCP-14579) and the Gram-negative bacteria were *Salmonella enterica* (NCCP-15756), *E. coli* (NCCP-14034), *Pseudomonas aeruginosa* (NCCP-16099), *Shigella dysenteriae* (NCCP-14746), *Klebsiella pneumoniae* (NCCP-14631), and *Enterobacter cloacae* (NCCP-14621). These bacteria were checked with the target bacteria (*E. coli* O157:H7, *E. coli*, and *E. coli* O145:H28) during the triplex PCR assay, and cross-reactivity was observed via PCR amplification with the specific length of the target band.

## 5. Conclusions

SNPs are the most common form of genetic variation, not only in *E. coli* but also in a wide variety of other bacterial species. SNP-based markers represent a target for PCR methods to easily and rapidly differentiate between different *E. coli* strains [51,66]. STEC pathogens still pose a major threat to public health, not only in developing countries but also in developed countries. To limit their spread and prevent infectious food-borne disease outbreaks, accurate and rapid diagnosis and classification of isolates are of great importance. SNPs are the most popular tool for the detection and study of genetic diversity and the phylogenetic analysis of any kind of genetic resource. This could be used in the medical sector for rapid and easy identification and typing of *E. coli*. In addition, the mutations can serve as phylogenetic markers for strain classification. Despite the importance of SNPs in our understanding of the diversity of *E. coli* populations, the research community is currently lacking a comprehensive database, even though multiple frontline laboratories in the USA and Canada have applied SNP analysis for the typing of STEC for clinical public health purposes [13]. SNP-based markers are also used for spatial epidemiology, typing, traceability, genetic information, determination, and genetic evolution analysis of food-borne pathogenic *E. coli*. Therefore, SNP-based studies promote food-borne disease outbreak monitoring and prevention, and the analysis and control of pathogenic *E. coli*. In this study, we used only *E. coli* obtained from wild-animal fecal samples for the evaluation of detection efficiency. However, further analysis and investigation should be conducted with a number of variable sources of *E. coli* for the evaluation of the efficiency of the developed SNP-based triplex marker assay.

## 6. Patents

Yung Chul Park, M.M. Rahman, and S.J. Lim. Development of multiplex PCR kit and detection of pathogenic *Escherichia coli* (*E. coli* O157:H7). Kangwon National University, Korea. Patent application No HP-17-076.

## Figures and Tables

**Table 1:** Information on natural SNP-containing primer sets based on whole-genome sequences (WGS) of *Escherichia coli* strains (*E. coli* O157 and non-O157).

P. Code ^C^	Forward Primer (5’–3′)	Reverse Primer (5’–3′)	Amplicon Size	Flanking Sequence with Ambiguous Code and Position of SNP of a Reference *E. coli* Genome ^a^	Alter Amino Acid-SNP Position in a Respective Gene-Amino Acid of Reference *E. coli* ^b^	Gene
01	GACGTTACAGCTGCCGGT	ACCCAACCAGTCGGCAAC	919	*thrB* (3196: C) GCTGGAAGGCVGTATCTCCGG.	(S/G)-396-R	Homoserine kinase (*thrB*)
02	TCGGCGGTCGCTTTATGG	CCACGGCTGCATAACCCA	669	*thrC* (4363: G) GTTAAACTCGDCTAACTCGAT.	(S/T)-630-A	Threonine synthase (*thrC*)
07	GCGAGCTGGGAGAACTGG	GATTCGCTGTACCGCCGG	674	*nhaR* (19293: C) AGAACGGCGABTTTTGATTCC.	(V/F)-579-L	Transcriptional activator protein (*nhaR*)
08	TGACTCGCCGTATGTGCC	TGCCCGGCAGATAAGTGC	839	*ileS* (22945: C) GAAGCCAGTTVACTGGTGCGT.	(N/D)-485-H; (V/F)-714-I; (Y/D)-747-N	Isoleucine--tRNA ligase (*ileS*)
				*ileS* (23104: A) CTCGCTGGTADTCTGGACCAC.		
				*ileS* (23137: A) TCTGCCTGCCDACCGCGCAAT.		
09	CGGCCTGGAAACCGCTAA	TCGGTTGATGCCACCCAC	852	*ileS* (23980: T) GCCGGACACAKTGGATGTATG.	V-1590-L	Isoleucine--tRNA ligase (*ileS*)
12	GGCATTAGAGGGCGTGCA	TGTCATACGGCTGGACGC	649	*dapB* (28751: G) TGTCTTTGCTVCCAATTTTAG.	(T/P)-378-A	4-hydroxy-tetrahydrodipicolinate (*dapB*)
14	TTGCTAAAGTGGCGGCGA	AGACGGATTCGCTTCGCA	713	*carB* (32142: T) CGCCGATGCGYTCCGTGCGGG.	L-1326-F	Carbamoyl-phosphate synthetase subunit beta (*carB*)
15	TATGCAGCCAGCCATCGG	AGATGTGGCACTTCCCGC	785	*caiC* (36991: A) AGGCGGCTGTVCCATCAACGT.	(G/A)-721-V	Carnitine-CoA ligase (*caiC*)
16	GGCGGTATACGGGAAGGC	CGTCTCCGGGGGATCTGA	702	*caiB* (39005: C) AACTTCCGCGMCCCATTCTGC.	V-1108-G	Carnitine-CoA transferase (*caiB*)
20	CCCAAGTTGCCCGGTCAT	GCACAGGGCTACGACGTT	738	*polB* (63783: A) GGTTTCGCGTVCATATTCCTG.	(G/A)-355-V	DNA polymerase II (*polB*)
				*polB* (63825: A) GCGCAGGTATMGCTCCTGCTG.	R-397-L	DNA polymerase II (*polB*)
21	CAAATGCCGCCACCGAAC	CGTCCGCCAGAACGCTAT	796	*polB* (65142: G) ATCGTAGTTGBCAAACCAGGC.	(G/D)-1714-A	DNA polymerase II (*polB*)
22	CCGATTGGCCTGCTTCCA	GCAGCTGTGGTCGGGATT	742	*araB* (69326: T) AGTCCAAGTGWCAGTGAACAG.	V-979-D	araB—Ribulokinase (*araB*)
23	TTGGCAGCGGCGAGTTAA	AAGCGTCGCATCAGGCAA	620	*yabI* (71824: G) CTTCCTGCCAKGGATTCTGGC.	W-474-G	Inner membrane protein (*yabI*)
24	CCATCTGGCGGGCGATAG	GGTGCTGGAGATGAGCGG	804	*thiP* (73115: G) CAGCACGCATRCAAAGGCCAG.	V-205-A	Thiamine transport system permease protein (*thiP*)
26	CAGTGGCGGCAGGAGTAC	CCCTGGGCATTGACCGAC	671	*leuD* (78863: G) CATAAACGCASGTTGTTTTGC.	L-16-P	3-isopropylmalate dehydratase subunit (*leuD*)

^a^ Reference (non-O157) genome of *Escherichia coli* str. K-12 substr. MG1655 (NC_000913) and ambiguous code indicate, B = C/G/T; D = A/G/T; H = A/C/T; V = A/C/G; W = A/T, S = C/G; M = A/C; K = G/T; R = A/G; Y = C/T; ^b^ the position of amino acid codes of respective genes of a reference *E. coli* str. K-12 substr. with changed amino acids due to SNP-based changes. The amino acid codes, S = Serine, G = Glycine, R = Arginine, T = Threonine, A = Alanine, V = Valine, F = Phenylalanine, D = Aspartate, N = Asparagine, Y = Tyrosine, Z = Glutamine, W = Tryptophan, P = Proline, M = Methionine, K = Lysine, L = Leucine, I = Isoleucine. ^C^ refers to primer code numbers (i.e., 01 ecoli, 02 ecoli, and 07 ecoli, and so on), which were originated from the designed multiple primers based on the encompassing SNPs of the reference *E. coli* genome.

**Table 2:** List of developed single-nucleotide polymorphism (SNP)-based primers based on five gene sequences.

Primer	Sequence (5′–3′) ^a^	Length (bp)	Amplicon Size (bp)	Gene Description
O.nhaR-1-F	TTGTTTGACGTTGGCGTGACT	21	397	Transcriptional activator protein (*nhaR*)
O.nhaR-1-R	CGGCATCATCAAACTCACCA	20		
O.nhaR-3-F	GCGTACTTAACGCCGCATTG	20	225	
O.nhaR-3-R	CCGGGAACGGTTTTTCTAGT	20		
O.thrB-3-F	TGTTCGGTGGTCGCGACG	18	337	Homoserine kinase (*thrB/C*)
O.thrB-3-R	CGTGAATGAAGCCAGCTAGA	20		
O.thrC-4-F	CCATTCTGACCGCGACCCCT	20	344	
O.thrC-4-R	AACCAGCTGGTTGCGCACC	19		
O.ileS1-4-F	TCTGGGCGTGCTGGGCAAT	19	488	Isoleucine–tRNA ligase (*ileS*)
O.ileS1-4-R	AGCGCAGCAGCTCAAGTTCT	20		
O.ileS1-3-F	ACAAAGGCGCGAAGCCAATT	20	391	
O.ileS1-3-R	GGATGGGTAAAGCGCAGTAA	20		
O.ileS2-1F	GATCATCTTCCGCGCAGCG	19	401	
O.ileS2-1R	CAACAACAGAAGAGTGAGTATAG	23		
O.ileS2-3-F	CGATCATCTTCCGCGCGCCG	20	376	
O.ileS2-3-R	GAGTCAAACCATACATCCAATTTG	24		
O.caiB-3-F	GCAAATTGCGGCGGGAGGGC	20	318	Carbamoyl-phosphate synthase large chain (*caiB*)
O.caiB-3-R	CTGCCTGATGCGACGTTAAT	20		
O.caiB-4-F	ATAACCAGTTTCGGGTTGCGC	21	296	
O.caiB-4-R	ATCGAAATCGCCGGACCGGTT	21		
O.polB-3-F	CAAGGGGCGACCGCGCTTCGA	21	262	DNA polymerase II (*polB*)
O.polB-3-R	GCTGGAAACCGTGCGCCCC	19		
O.polB-4-F	TAATGGTGCCGCGGTTCTGG	20	232	
O.polB-4-R	CTTTACCTGCGTATCTTCAGT	21		

^a^ Red color indicates natural SNP and blue color indicates artificial SNP with transition or transversion mutated.

**Table 3:** Selection of SNP primers with the target band pattern of serotype-specific *E. coli* strains.

Primer	*E. coli* O157:H7 (ATCC-95150)	*E. coli* O157:H7 (NCCP-15739)	*E. coli* (KVCC-BA1800069)	*E. coli* O145:H28 (KVCC-BA1800090)
O.nhaR1	√	√		√
O.ileS2-1	√	√		
O.thrB-3			√	
O.nhaR-3			√	
O.ileS1-3			√	
O.ileS2-3	+		√	√
O.caiB-3		++	√	
O.polB-3			+	√
O.thrC-4				√
O.ileS1-4				√
O.caiB-4			√	√
O.polB-4				√

“√” indicates a positive target band, “+” indicates an off-target single band, and “++” indicates the off-target double band.

**Table 4:** Information on triplex SNP primers (O1) and their target strains.

Primer Name	Sequence (5′–3′)	Length (bp)	Amplicon Size (bp)	Target Strains
O.ileS2-1F	GATCATCTTCCGCGCAGCG	19	401	*E. coli* O157:H7 (ATTC-95150; NCCP-15739)
O.ileS2-1R	CAACAACAGAAGAGTGAGTATAG	23		
O.thrB-3-F	TGTTCGGTGGTCGCGACG	18	337	*E. coli* (KVCC-BA1800069)
O.thrB-3-R	CGTGAATGAAGCCAGCTAGA	20		
O.polB-4-F	TAATGGTGCCGCGGTTCTGG	20	232	*E. coli* O145:H28 (KVCC-BA1800090)
O.polB-4-R	CTTTACCTGCGTATCTTCAGT	21		

## Data Availability

In this study, the genetic sequence data used were deposited into the NCBI with the following GenBank accession numbers (OL589326-OL589363). Moreover, the data will be available on request to the corresponding author.

## Supplementary Materials

The followings are available online at https://www.mdpi.com/article/10.3390/pathogens11020115/s1, Figure S1: The alignment of 01 ecoli_F/R primer amplified gene sequences (homoserine kinase-thrB) of four target *E. coli* strains. Primer pair is indicated by underline and italicized region, natural SNP base is marked by a square shape, and artificial mutated SNP base is marked by a black shaded square shape. Figure S2: The alignment of 08 ecoli_F/R primer amplified gene sequences (isoleucine-tRNA transfer ligase, ileS) of four target *E. coli* strains. Primer pair is indicated by underline. The natural SNP base is marked by a square shape, and artificial mutated SNP base is marked by a black shaded square shape. Here two primer pairs are presented (iles “1-4-F/R” and “iles3F/R”) Figure S3: PCR amplification of *Escherichia coli* strain-specific triplex primer set (O1) with laboratory-isolated *E. coli*. Table S1: The position of single nucleotide polymorphism (SNP) bases, accession numbers of *E. coli* strains for reference and comparison. Table S2: The amplification of target *Escherichia coli* strains with 15 flanking primers, their sequences, and alignment, and SNP-based primer design on the aligned sequences. Table S3: Information on SNP-encompassing primer amplification success rate. Table S4: The successful amplification of all SNP-encompassing primers with target *E. coli* strains, their gene bank accession numbers, with similarity tests. Table S5: Information on the triplex SNP-based PCR marker (O1) for serotype-specific *E. coli* detection.
