# Supplementary material for: Development of Single Nucleotide Polymorphism (SNP)-Based Triplex PCR Marker for Serotype-specific Escherichia coli Detection
Source: Pathogens. 2022 Jan 19;11(2):115. doi: 10.3390/pathogens11020115 (PMC8874422; doi:10.3390/pathogens11020115)
Supplement: Supplementary file 1 [file pathogens-11-00115-s001.zip › Figure S3..pptx]

## Slide 1
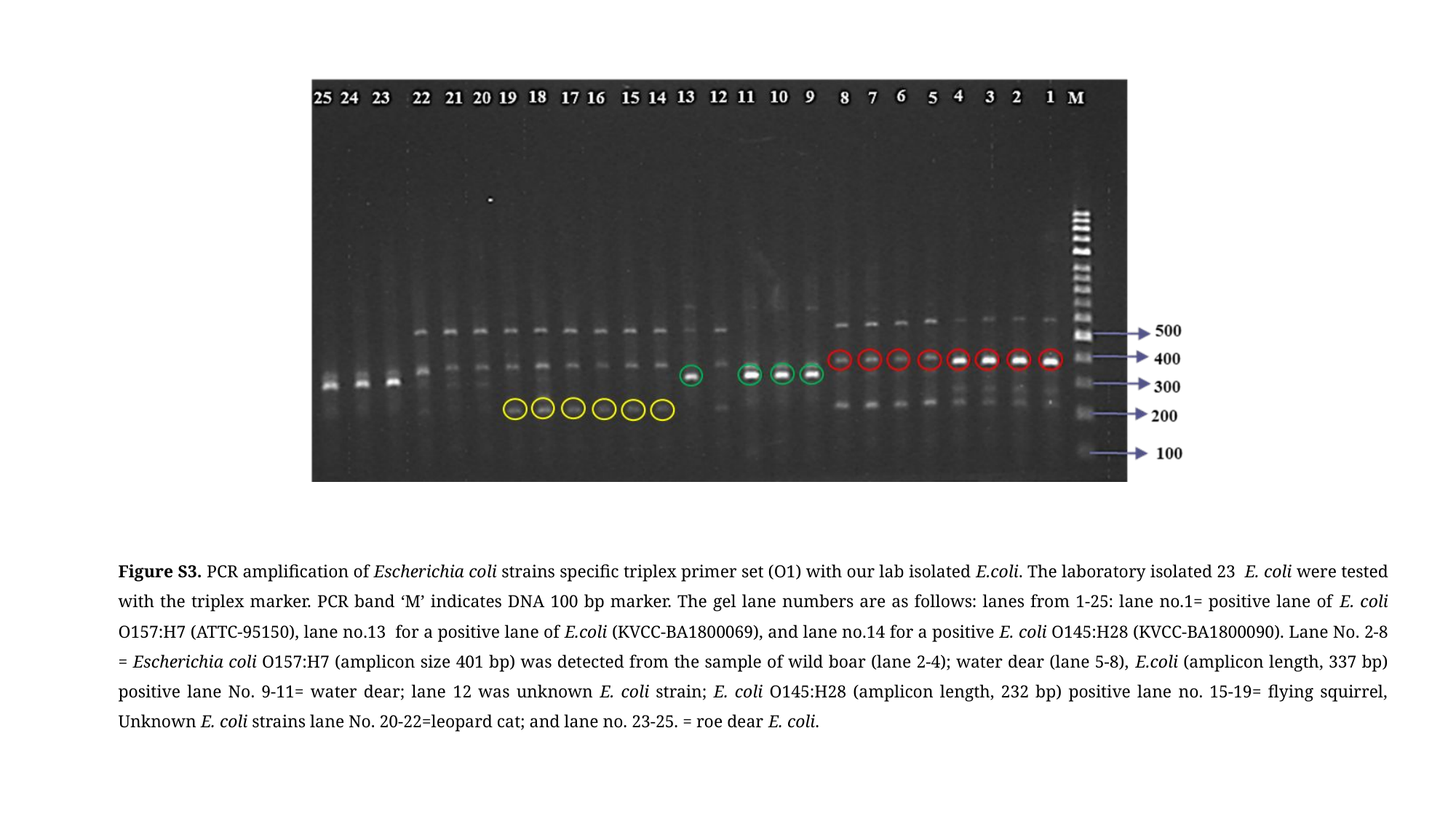

Figure S3. PCR amplification of Escherichia coli strains specific triplex primer set (O1) with our lab isolated E.coli. The laboratory isolated 23 E. coli were tested with the triplex marker. PCR band ‘M’ indicates DNA 100 bp marker. The gel lane numbers are as follows: lanes from 1-25: lane no.1= positive lane of E. coli O157:H7 (ATTC-95150), lane no.13 for a positive lane of E.coli (KVCC-BA1800069), and lane no.14 for a positive E. coli O145:H28 (KVCC-BA1800090). Lane No. 2-8 = Escherichia coli O157:H7 (amplicon size 401 bp) was detected from the sample of wild boar (lane 2-4); water dear (lane 5-8), E.coli (amplicon length, 337 bp) positive lane No. 9-11= water dear; lane 12 was unknown E. coli strain; E. coli O145:H28 (amplicon length, 232 bp) positive lane no. 15-19= flying squirrel, Unknown E. coli strains lane No. 20-22=leopard cat; and lane no. 23-25. = roe dear E. coli.
