# Supplementary material for: Development of Single Nucleotide Polymorphism (SNP)-Based Triplex PCR Marker for Serotype-specific Escherichia coli Detection
Source: Pathogens. 2022 Jan 19;11(2):115. doi: 10.3390/pathogens11020115 (PMC8874422; doi:10.3390/pathogens11020115)
Supplement: Supplementary file 1 [file pathogens-11-00115-s001.zip › Table S2.pdf]

Supplementary Table S2. The amplification of target *Escherichia coli* strains with 15 flanking primers, their sequences, and alignment, SNPs-based primer design on the aligned sequences

NO	*Name of E. coli*	Catalog No.	Source
**1**	*E. coli* O157:H7	ATCC-95150	American type culture collection
**2**	*E. coli* O157:H7	NCCP-15739	National culture collection of pathogens
**3**	*E. coli*	KVCC-BA1800069	Korean veterinary culture collection
**4**	*E. coli* O145:H28	KVCC-BA1800090	Korean veterinary culture collection

The information of target three different category four *Escherichia coli* strains

1-ecoli primer set: Gene Name: Homoserine kinase *(thrB)*

01- ecoli-F	GACGTTACAGCTGCCGGT
01- ecoli-R	ACCCAACCAGTCGGCAAC

>01-ecoli-1_F

CGGGTCATACAGTATGAGTGTACTCTCATGGAGTTAGGAGTCTGACATGGTTAAAGTTTATGCCCCGGCTTCCAGTGCCA

ATATGAGCGTCGGGTTTGATGTGCTCGGGGCGGCGGTGACACCCGTTGATGGTGCATTGCTCGGAGATGTAGTCACGGTT

GAGTCGGCAGAGACATTCAGTCTCAACAACCTCGGACGCTTTGCCGATAAGCTGCCGTCAGAACCACGGGAAAATATCGT

TTATCAGTGCTGGGAGCGTTTTTGCCAGGAGCTGGGCAAGCAAATTCCAGTGGCGATGACTCTGGAAAAGAATATGCCGA

TCGGTTCGGGCTTAGGCTCCAGCGCCTGTTCGGTGGTCGCGGCTCTGATGGCGATGAATGAACACTGCGGCAAGCCGCTT

AATGACACCCGTTTGCTGGCTTTGATGGGCGAGCTGGAAGGACGAATCTCCGGCAGCATTCATTACGACAACGTGGCACC

GTGTTTTCTTGGTGGTATGCAGTTGATGATCGAAGAAAACGACATCATCAGCCAGCAAGTGCCAGGGTTTGATGAGTGGC

TGTGGGTGCTGGCGTATCCGGGGATTAAAGTCTCGACGGCAGAAGCCCGGGCTATTTTACCGGCGCAGTATCGCCGCCAG

GATTGCATTGCGCACGGGCGACACTTGGCAGGCTTCATTCACGCCTGCTATTCCCGTCAGCCTGAGCTTGCCGCGAAGCT

GATGAAAGATGTTATCGCAGAACCCTACCGTGAACGGTTACTGCCTGGCTTCCGGCAGGCGCGGCAGGCGGTCGCGGAAA

TCGGCGCGGTAGCGAGCGGTATCTCCGGCTCCGGCCCGACCTTGTTTGCTCTGTGTGACAAGCCGGATACCGCCCAGCGC

GTTGCGCTTTTGGTTGGGGTATTCCGGGCTTTGTCAACAAAAGAAAACAGGGTCGGGCCGGAGCCGAAATTCCGCTAGCT

TACCGCGCTATTTTCGAACGCCTGCCGCGCCGTCGGATACCAGCGTAAGCAGTTGCAGGTTTAGGGGTTCTGCAAATAAC

TCCTGTTATCACCTTGCGACGCTCAGCTGTACGGTTATTGCCATAGCTGTCAATTAATCTGCCTGTGTTGCCTGCATGTA

CTCTGGCGCCATTCTGGCAATAAGTGAGCGAGTCTCTGCTAGGACTCGCATTCTTGAGACTGAGA

>01-ecoli-2_F

GAGGCAAGTTAGTACCTCTCATGGAGTAGGAGTCTGACATGGTTAAAGTTTATGCCCCGGCTTCCAGTGCCAATATGAGC

GTCGGGTTTGATGTGCTCGGGGCGGCGGTGACACCCGTTGATGGTGCATTGCTCGGAGATGTAGTCACGGTTGAGTCGGC

AGAGACATTCAGTCTCAACAACCTCGGACGCTTTGCCGATAAGCTGCCGTCAGAACCACGGGAAAATATCGTTTATCAGT

GCTGGGAGCGTTTTTGCCAGGAGCTGGGCAAGCAAATTCCAGTGGCGATGACTCTGGAAAAGAATATGCCGATCGGTTCG

GGCTTAGGCTCCAGCGCCTGTTCGGTGGTCGCGGCTCTGATGGCGATGAATGAACACTGCGGCAAGCCGCTTAATGACAC

CCGTTTGCTGGCTTTGATGGGCGAGCTGGAAGGACGAATCTCCGGCAGCATTCATTACGACAACGTGGCACCGTGTTTTC

TTGGTGGTATGCAGTTGATGATCGAAGAAAACGACATCATCAGCCAGCAAGTGCCAGGGTTTGATGAGTGGCTGTGGGTG

CTGGCGTATCCGGGGATTAAAGTCTCGACGGCAGAAGCCCGGGCTATTTTACCGGCGCAGTATCGCCGCCAGGATTGCAT

TGCGCACGGGCGACACTTGGCAGGCTTCATTCACGCCTGCTATTCCCGTCAGCCTGAGCTTGCCGCGAAGCTGATGAAAG

ATGTTATCGCAGAACCCTACCGTGAACGGTTACTGCCTGGCTTCCGGCAGGCGCGGCAGGCGGTCGCGGAAATCGGCGCG

GTAGCGAGCGGTATCTCCGGCTCCGGCCCGACCTTGTTTGCTCTGTGTGACAAGCCGGATACCGCCCAGCGCGTTGCGCT

TTTGGTTGGGTAATTCGGGCTTTGTTCAACAAAGCAACATGGTCGGGGCGGACCGGAAATCCCGCTTGCTACCGCGCCAT

TTCCGACGCCTGCCGCCCCCGTCCGAAGACCAGCGGATACATTTCAGAGGTAAGGGGGTTGCTCTATATAACATTCTATT

ATCAAACTGCGTGCTGCTCGATGCTGTACATGGAATTATTACGCGTGTC

>01-ecoli-3_F

CGGCTTGGTAGTACTCTCATGGAGTTAGGAGTCTGACATGGTTAAAGTTTATGCCCCGGCTTCCAGTGCCAATATGAGCG

TCGGGTTTGATGTGCTCGGGGCGGCGGTGACACCTGTTGATGGTGCATTGCTCGGAGATGTAGTCACGGTTGAGGCGGCA

GAGACATTCAGTCTCAACAACCTCGGACGCTTTGCCGATAAGCTGCCGTCAGAACCACGGGAAAATATCGTTTATCAGTG

CTGGGAGCGTTTTTGCCAGGAGCTTGGCAAGCAAATTCCAGTGGCGATGACTCTGGAAAAGAATATGCCAATCGGTTCGG

GCTTAGGCTCCAGCGCCTGTTCGGTGGTCGCGGCGCTGATGGCGATGAATGAACACTGTGGCAAGCCGCTTAATGACACT

CGTTTGCTGGCTTTGATGGGCGAGCTGGAAGGACGAATCTCCGGCAGCATTCATTACGACAACGTGGCACCGTGTTTTCT

TGGTGGTATGCAGTTGATGATCGAAGAAAACGACATCATCAGCCAGCAAGTGCCAGGGTTTGATGAGTGGCTGTGGGTGC

TGGCGTATCCGGGGATTAAAGTCTCGACGGCAGAAGCCAGGGCTATTTTACCGGCGCAGTATCGCCGCCAGGATTGCATT

GCGCACGGGCGACATCTGGCTGGCTTCATTCACGCCTGCTATTCCCGTCAGCCTGAGCTTGCCGCGAAGCTGATGAAAGA

TGTTATCGCTGAACCCTACCGTGAACGGTTACTGCCTGGCTTCCGGCAGGCGCGGCAGGCGGTCGCGGAAATCGGCGCGG

TAGCGAGCGGTATCTCCGGCTCCGGCCCGACCTTGTTCGCTCTATGTGACAAGCCGGATACCGCCCAGCGCGTTGCGCTT

GTGGTTTGGGGTAAATCCGGGCTTGTCACATAGAGCGAAAAGGTTCGGGCGGAGCGGAGATTCCGCTTGCTTACGCGCGA

TTTCGGTACGCTGCCGCGCCGGCTGATAGACAGGAGCTTATACCCTTTCGAGGTGGGGTTTAGAATAACATCTTTTACCA

CTTTGCGCTCAGTCTAGTCGTGTGACGGAATTGACAGGGCGGTATTGAATCGACTAATTGTCGGGCCATGTCACTGGCAC

AATTCGCTAAGTCTGCCTCGTCCGATAACTGAACTGCTAGACCATGA

>01-ecoli-4_F

CAGCCTTGCTGTACTCTCATGGAGTTAGGAGTCTGACATGGTTAAAGTTTATGCCCCGGCTTCCAGTGCCAATATGAGCG

TCGGGTTTGATGTGCTCGGGGCGGCGGTGACACCCGTTGATGGTGCATTGCTCGGAGATGTAGTAACGGTTGAGGCGGCA

GAGACATTCAGTCTCAACAACCTCGGACGCTTTGCCGATAAGCTGCCGTCAGAACCACGGGAAAATATCGTTTATCAGTG

CTGGGAGCGTTTTTGCCAGGAGCTGGGCAAGCAAATTCCAGTGGCGATGACTCTGGAAAAGAATATGCCGATCGGTTCGG

GCTTAGGCTCCAGCGCCTGTTCGGTGGTCGCGGCGCTGATGGCGATGAATGAACACTGTGGCAAGCCGCTTAATGACACT

CGTTTGCTGGCTTTGATGGGCGAGCTGGAAGGACGAATCTCCGGCAGCATACATTACGACAACGTGGCACCGTGTTTTCT

TGGTGGTATGCAGTTGATGATCGAAGAAAACGACATCATCAGCCAGCAAGTGCCTGGGTTTGATGAGTGGCTGTGGGTGC

TGGCGTATCCGGGGATTAAAGTCTCGACGGCAGAAGCCCGGGCTATTTTACCGGCGCAGTATCGCCGCCAGGATTGCATT

GCGCACGGGCGACACTTGGCAGGCTTCATTCACGCCTGCTATTCCCGTCAGCCTGAGCTTGCCGCGAAGCTGATGAAAGA

TGTTATCGCTGAACCCTACCGTGAACGGTTACTGCCTGGCTTCCGGCAGGCGCGGCAGGCGGTCGCGGAAATCGGCGCGG

TAGCGAGCGGTATCTCCGGCTCCGGCCCGACCTTGTTCGCTCTGTGTGACAAGCCGGATACCGCCCAGCGCGTTGCGTTC

TGGGTGTTGGTGGGGGTAACTCCGGGCTTGGTCACACAAAAGCGAAAAAGGGTCGGGGCCGGAAGCCGGGAAAATCCCGC

TCGCTACCGCGCTAATTTTCGGAACGGCCTGCCGCGCCGGCCGGAAGACCGGCGTTAACGCTTTCACGGTTGGGGGGTTC

AGCAATACCATTCTTTTCACTAACCTTGCGCAGGCTCAAGCTGAACGGGAAAATGACAGGACTAAAATGTGAAGACTCGT

GCCAGATGTGGCGTGCCCAGATGTTACATCTGACGACAATCGCGTGCTATAAATGAGCGGTGCGTATCTGCCCGCTAGTA

AC

```
                      10        20        30        40        50        60        70        80
              ....|....|....|....|....|....|....|....|....|....|....|....|....|....|....|....|....|...
01-ecoli-1_F  CTCTCATGGAGTTAGGAGTCTGACATGGTTAAAGTTTATGCCCCGGCTTCCAGTGCCAATATGAGCGTCGGGTTTGATGTGCTCGGGG
01-ecoli-2_F  ............-...........................................................................
01-ecoli-3_F  ........................................................................................
01-ecoli-4_F  ........................................................................................

                     110       120       130       140       150       160       170       180
              ....|....|....|....|....|....|....|....|....|....|....|....|....|....|....|....|....|...
01-ecoli-1_F  CCGTTGATGGTGCATTGCTCGGAGATGTAGTCACGGTTGAGTCGGCAGAGACATTCAGTCTCAACAACCTCGGACGCTTTGCCGATAA
01-ecoli-2_F  ........................................................................................
01-ecoli-3_F  .T.......................................G..............................................
01-ecoli-4_F  ...............................A.........G..............................................

                     210       220       230       240       250       260       270       280
              ....|....|....|....|....|....|....|....|....|....|....|....|....|....|....|....|....|...
01-ecoli-1_F  ACCACGGGAAAATATCGTTTATCAGTGCTGGGAGCGTTTTTGCCAGGAGCTGGGCAAGCAAATTCCAGTGGCGATGACTCTGGAAAAG
01-ecoli-2_F  ........................................................................................
01-ecoli-3_F  ...................................................T....................................
01-ecoli-4_F  ........................................................................................

                     310       320       330       340       350       360       370       380
              ....|....|....|....|....|....|....|....|....|....|....|....|....|....|....|....|....|...
01-ecoli-1_F  GGTTCGGGCTTAGGCTCCAGCGCCTGTTCGGTGGTCGCGGCTCTGATGGCGATGAATGAACACTGCGGCAAGCCGCTTAATGACACCC
01-ecoli-2_F  ........................................................................................
01-ecoli-3_F  .........................................G.......................T....................T.
01-ecoli-4_F  .........................................G.......................T....................T.

                     410       420       430       440       450       460       470       480
              ....|....|....|....|....|....|....|....|....|....|....|....|....|....|....|....|....|...
01-ecoli-1_F  TGATGGGCGAGCTGGAAGGACGAATCTCCGGCAGCATTCATTACGACAACGTGGCACCGTGTTTTCTTGGTGGTATGCAGTTGATGAT
01-ecoli-2_F  ........................................................................................
01-ecoli-3_F  ........................................................................................
01-ecoli-4_F  .....................................A..................................................

                     510       520       530       540       550       560       570       580
              ....|....|....|....|....|....|....|....|....|....|....|....|....|....|....|....|....|...
01-ecoli-1_F  CATCATCAGCCAGCAAGTGCCAGGGTTTGATGAGTGGCTGTGGGTGCTGGCGTATCCGGGGATTAAAGTCTCGACGGCAGAAGCCCGG
01-ecoli-2_F  ........................................................................................
01-ecoli-3_F  .....................................................................................A..
01-ecoli-4_F  .....................T..................................................................

                     610       620       630       640       650       660       670       680
              ....|....|....|....|....|....|....|....|....|....|....|....|....|....|....|....|....|...
01-ecoli-1_F  GCGCAGTATCGCCGCCAGGATTGCATTGCGCACGGGCGACACTTGGCAGGCTTCATTCACGCCTGCTATTCCCGTCAGCCTGAGCTTG
01-ecoli-2_F  ........................................................................................
01-ecoli-3_F  .........................................TC....T........................................
01-ecoli-4_F  ........................................................................................

                     710       720       730       740       750       760       770       780
              ....|....|....|....|....|....|....|....|....|....|....|....|....|....|....|....|....|...
01-ecoli-1_F  TGAAAGATGTTATCGCAGAACCCTACCGTGAACGGTTACTGCCTGGCTTCCGGCAGGCGCGGCAGGCGGTCGCGGAAATCGGCGCGGT
01-ecoli-2_F  ........................................................................................
01-ecoli-3_F  ................T.......................................................................
01-ecoli-4_F  ................T.......................................................................

                     810       820       830       840       850       860
              ....|....|....|....|....|....|....|....|....|....|....|....|....|..
01-ecoli-1_F  CTCCGGCTCCGGCCCGACCTTGTTTGCTCTGTGTGACAAGCCGGATACCGCCCAGCGCGTTGCGCTT
01-ecoli-2_F  ...................................................................
01-ecoli-3_F  ........................C.....A....................................
01-ecoli-4_F  ........................C.......................................T.C
```

O.thrB-3-F: TGT TCG GTG GTC GCG ACG

O.thrB-3-R : CGT GAA TGA AGC CAG CTA GA

02-ecoli-marker set; Gene Name: Homoserine kinase (*thrC*)

02-ecoli F : TCGGCGGTCGCTTTATGG	
02-ecoli-R CCACGGCTGCATAACCCA	

>02-ecoli-1_F

GGACCCCATTTGCGGCGTAGCAGTGACCATTCTGACCGCGACCTCCGGTGATACCGGAGCGGCAGTGGCTCATGCTTTCT

ACGGTTTACCGAATGTGAAAGTGGTTATCCTTTATCCACGAGGCAAAATCAGTCCACTGCAAGAAAAACTGTTCTGTACA

TTGGGCGGCAATATCGAAACTGTTGCCATCGACGGCGATTTCGATGCCTGTCAGGCGCTGGTGAAGCAGGCGTTTGATGA

TGAAGAGCTGAAAGTGGCGCTGGGGTTAAACTCAGCTAACTCGATTAACATTAGCCGGTTGCTGGCGCAGATTTGCTACT

ACTTTGAAGCAGTTGCGCAGCTGCCGCAGGAAGCGCGCAACCAGCTGGTTGTCTCGGTGCCAAGCGGAAACTTCGGCGAT

TTGACGGCGGGTCTGCTGGCGAAGTCACTCGGTCTGCCGGTGAAACGTTTTATTGCTGCGACCAACGTGAACGATACCGT

GCCACGTTTCCTGCATGACGGTCAGTGGTCACCCAAAGCGACTCAGGCGACGTTATCCAACGCGATGGACGTGAGTCAGC

CGAACAACTGGCCGCGTGTGGAAGAGTTGTTCCGCCGCAAATCTGGCAACTGAAAGAGCTGGGTTAGCCAAGCCGTGGAA

GGATACCCCCTTGGAAATTTGGCAATCACTTAGATTGGTTTGTTCTGTAGTGAGTGTTTTTTTAATGTTAATTACTTTAT

GTTATTCGCTCTTCCCGGCCCCCGCTCGGA

>02-ecoli-2_F

GGATCATTGCGGGCGTAGCAGTGACCATTCTGACCGCGACCTCCGGTGATACCGGAGCGGCAGTGGCTCATGCTTTCTAC

GGTTTACCGAATGTGAAAGTGGTTATCCTTTATCCACGAGGCAAAATCAGTCCACTGCAAGAAAAACTGTTCTGTACATT

GGGCGGCAATATCGAAACTGTTGCCATCGACGGCGATTTCGATGCCTGTCAGGCGCTGGTGAAGCAGGCGTTTGATGATG

AAGAGCTGAAAGTGGCGCTGGGGTTAAACTCAGCTAACTCGATTAACATTAGCCGGTTGCTGGCGCAGATTTGCTACTAC

TTTGAAGCAGTTGCGCAGCTGCCGCAGGAAGCGCGCAACCAGCTGGTTGTCTCGGTGCCAAGCGGAAACTTCGGCGATTT

GACGGCGGGTCTGCTGGCGAAGTCACTCGGTCTGCCGGTGAAACGTTTTATTGCTGCGACCAACGTGAACGATACCGTGC

CACGTTTCCTGCATGACGGTCAGTGGTCACCCAAAGCGACTCAGGCGACGTTATCCAACGCGATGGACGTGAGTCAGCCG

AACAACTGGCCGCGTGTGGAAGAGTTGTTCCGCCGCAAAATCTGGCAACTGAAAGAGCTGGGTTAGCCAAGCCGTGGA

>02-ecoli-3_F

GTGAGCATTTGCGGCGTAGCCAGTGACCATTCTGACCGCGACCTCCGGTGATACCGGAGCGGCAGTGGCTCATGCTTTCT

ACGGTTTACCGAATGTGAAAGTGGTTATCCTCTATCCACGAGGCAAAATCAGTCCACTGCAAGAAAAACTGTTCTGTACG

TTGGGCGGCAATATCGAAACTGTTGCCATCGACGGCGATTTCGATGCCTGTCAGGCGCTGGTGAAGCAGGCGTTTGATGA

TGAAGAACTGAAAGTGGCGCTGGGGTTAAACTCAGCTAACTCGATTAACATCAGCCGTTTGCTGGCGCAGATTTGCTACT

ACTTTGAAGCTGTTGCGCAGCTGCCGCAGGAAGCGCGCAACCAGCTGGTTGTCTCGGTGCCAAGCGGAAACTTCGGCGAT

TTGACGGCGGGTCTGCTGGCGAAGTCACTCGGTCTGCCGGTGAAACGTTTTATTGCTGCGACCAACGTGAACGATACCGT

GCCACGTTTCCTGCACGACGGTCAGTGGTCACCCAAAGCGACTCAGGCGACGTTATCCAACGCGATGGACGTGAGTCAGC

CGAACAACTGGCCGCGTGTGGAAGAGTTGTTCCGCCGCAAAATCTGGCAACTGAAAGAGCTGGGTTAGCCAGCCGTGGA

>02-ecoli-4_F

GGACCCTTGCGGCGTAGCCAGTGACCATTCTGACCGCGACCTCTGGTGATACCGGAGCGGCAGTGGCTCATGCTTTCTAC

GGTTTACCGAATGTGAAAGTGGTTATCCTCTATCCACGAGGCAAAATCAGTCCACTGCAAGAAAAACTGTTCTGTACATT

GGGCGGCAATATCGAAACTGTTGCCATCGACGGCGATTTCGATGCCTGTCAGGCGCTGGTGAAGCAGGCGTTTGATGATG

AAGAGCTGAAAGTGGCGCTGGGGTTAAACTCAGCTAACTCGATTAACATCAGCCGGTTGCTGGCGCAGATTTGCTACTAC

TTTGAAGCTGTTGCGCAGCTGCCGCAGGAGGCGCGCAACCAGCTGGTTGTCTCGGTGCCAAGCGGAAACTTCGGCGATTT

GACGGCGGGTCTGCTGGCGAAGTCACTCGGTCTGCCGGTGAAACGTTTTATTGCTGCGACCAACGTGAACGATACCGTGC

CACGTTTCCTGCACGACGGTCAGTGGTCACCCAAAGCGACTCAGGCGACGTTATCCAACGCGATGGACGTGAGTCAGCCG

AACAACTGGCCGCGTGTGGAAGAGTTGTTCCGCCGCAAATCTGGCAACTGAAAGAGCTGGGTTAGCCAAGCCCGTGAGAG

AGGTTACCCACTTTCACATTCGGTAAACCGTTACAAAGTATGTGGCACTGTTCGTCTTGTTTTGGAGAAACTTC

```
                      10        20        30        40        50        60        70        80
              ....|....|....|....|....|....|....|....|....|....|....|....|....|....|....|....|....|...
02-ecoli-1_F  TGCGGCGTAGC-AGTGACCATTCTGACCGCGACCTCCGGTGATACCGGAGCGGCAGTGGCTCATGCTTTCTACGGTTTACCGAATGTG
02-ecoli-2_F  GCG........-............................................................................
02-ecoli-3_F  ...........C............................................................................
02-ecoli-4_F  ...........C........................T...................................................

                     110       120       130       140       150       160       170       180
              ....|....|....|....|....|....|....|....|....|....|....|....|....|....|....|....|....|...
02-ecoli-1_F  CTTTATCCACGAGGCAAAATCAGTCCACTGCAAGAAAAACTGTTCTGTACATTGGGCGGCAATATCGAAACTGTTGCCATCGACGGCG
02-ecoli-2_F  ........................................................................................
02-ecoli-3_F  ..C...............................................G.....................................
02-ecoli-4_F  ..C.....................................................................................

                     210       220       230       240       250       260       270       280
              ....|....|....|....|....|....|....|....|....|....|....|....|....|....|....|....|....|...
02-ecoli-1_F  GTCAGGCGCTGGTGAAGCAGGCGTTTGATGATGAAGAGCTGAAAGTGGCGCTGGGGTTAAACTCAGCTAACTCGATTAACATTAGCCG
02-ecoli-2_F  ........................................................................................
02-ecoli-3_F  .....................................A............................................C.....
02-ecoli-4_F  ..................................................................................C.....

                     310       320       330       340       350       360       370       380
              ....|....|....|....|....|....|....|....|....|....|....|....|....|....|....|....|....|...
02-ecoli-1_F  GATTTGCTACTACTTTGAAGCAGTTGCGCAGCTGCCGCAGGAAGCGCGCAACCAGCTGGTTGTCTCGGTGCCAAGCGGAAACTTCGGC
02-ecoli-2_F  ........................................................................................
02-ecoli-3_F  .....................T..................................................................
02-ecoli-4_F  .....................T....................G.............................................

                     410       420       430       440       450       460       470       480
              ....|....|....|....|....|....|....|....|....|....|....|....|....|....|....|....|....|...
02-ecoli-1_F  GGTCTGCTGGCGAAGTCACTCGGTCTGCCGGTGAAACGTTTTATTGCTGCGACCAACGTGAACGATACCGTGCCACGTTTCCTGCATG
02-ecoli-2_F  ........................................................................................
02-ecoli-3_F  ......................................................................................C.
02-ecoli-4_F  ......................................................................................C.

                     510       520       530       540       550       560       570       580
              ....|....|....|....|....|....|....|....|....|....|....|....|....|....|....|....|....|...
02-ecoli-1_F  CACCCAAAGCGACTCAGGCGACGTTATCCAACGCGATGGACGTGAGTCAGCCGAACAACTGGCCGCGTGTGGAAGAGTTGTTCCGCCG
02-ecoli-2_F  ........................................................................................
02-ecoli-3_F  ........................................................................................
02-ecoli-4_F  ........................................................................................

                     610       620
              ....|....|....|....|....|
02-ecoli-1_F  ACTGAAAGAGCTGGGTTAGCCAAGC
02-ecoli-2_F  .........................
02-ecoli-3_F  ......................-..
02-ecoli-4_F  .........................
```

O-thrC-4-F : CCA TTC TGA CCG CGA CCC CT

O-thrC-4-R : AAC CAG CTG GTT GCG CAC C

07-ecoli-marker set, Gene Name: Transcriptional activator protein (*nhaR*)

07- ecoli-F	GCGAGCTGGGAGAACTGG
07- ecoli-R	GATTCGCTGTACCGCCGG

```
>07-ecoli-1_F

GTCAGAGCTGCTGTAGACTTGAGCAGGAATGCTGGATATCGTGAACTATCGCAAAGAATCCAATTTATTGTTTGACGTTG

GCGTGGCTGATGTACTTTCCAAACGTCTGGTCAGTAGCGTGCTGAATGCTGCAGTAGTAGAAGGCGAGCCGATTCATCTT

CGCTGCTTCGAATCCACCCACGAAATGCTGCTGGAGCAATTAAGCCAGCATAAACTGGATATGATCATTTCCGACTGTCC

GATAGATTCTACGCAGCAGGAAGGCCTGTTCTCCGTGAGAATTGGCGAATGTGGCGTGAGTTTCTGGTGTACAAATCCAC

CGCCGGAAAAACCGTTCCCAGCTTGTCTTGAAGAACGGCGGCTTTTGATTCCTGGGCGACGTTCAATGTTAGGGCGCAAA

TTACTTAACTGGTTTAACTCCCAGGGATTAAACGTAGAAATCCTTGGCGAGTTTGATGATGCCGCTTTGATGAAAGCTTT

TGGTGCGATGCACAATGCAATCTTCGTTGCCCCAACGCTTTATGCATATGACTTTTATGCCGATAAAACTGTCGTAGAAA

TTGGTCGCGTCGAGAATGTGATGGAAGAGTACCATGCTATTTTTGCTGAGCGGATGATTCAGCACCCGGCGGTCCGGCGA

ATCAGAGCGCCTGGCGCACGGGGAAGCGGACGTCACTTTTTCGGAGGGACAGCGTAATCAAAGTTTTTTGGGCTTAAAAT

TCAAATGCTTAAGCGCTTGGGGCCTAATGCATGTGCATCGCACAAAGCTTTTCTAAAGCGCTCTTCCATGCGCGGATTTT

CTTTTTTACCCTGCGAGTTAACCTTATATGGGCTGCCACTGTAGTGCGCAGCACATAAACACTTCTCTCAACATAGTAGG

GTTCTGTCGTGAATTATACTAAACTCGACCTCCGCTGC

>07-ecoli-2_F

CGCAGCATGTTACGCTGAGCAGGAGATGCTGGATATCGTCATTACCGCAAAGAATCCACCTGTTGTTTGATGTCGGCGTC

GCCGATGCTTTATCCAAGCGTCTGGTCAGCAGCGTTCTGGATGCTGCGGTGGTGGTGGACGAACCCATACATCTGCGTTG

CTTCGAGTCCACGCACGAAATGTTGCTCGAACAGCTCAGCCAGCACAAGCTGGATATGATTATCTCCGACTGCCCGATAG

ATTCCACGCAGCAGGAGGGGCTATTCTCAGTCAAAATCGGCGAATGCAGCGTCAGTTTCTGGTGTACGAATCCGTTTCCT

GAAAAAGCGTTCCCTGCCTGCCTGGAAGAGCGACGGTTGCTGGTGCCGGGACGGCGCTCAATGCTGGGGCGTAAGTTGTT

GAACTGGTTTAATTCACAGGGGTTGCAGGTTGAGATCCTCGGTGAATTTGATGATGCGGCCTTAATGAAGGCGTTTGGCG

CGACGCACGATGCGATTTTTGTGGCGCCGAGCCTTTATTCGCTGGATTTTTATGCCGATGAGTCGGTAATAGAGATTGGG

CGCGTGGAGAATGTCATGGAAGAATATCATGCTATTTTTGCTGAGAGAATGATCCAGCACCCGGCGGTCCAGCGAATCAA

G

>07-ecoli-3_F

TGCCGCTTACTGTCCCTTAGCCAGGAATGCTGGATATTGTGAACTATCGCAAAGAATCCAATTTATTGTTTGACGTTGGT

GTGGCTGATGCACTTTCCAAACGCCTGGTCAGTAGCGTACTTAACGCCGCAGTGGTAGAAGGCGAGCCCATTCATCTTCG

CTGCTTCGAATCCACCCACGAAATGCTGCTGGAGCAATTAAGTCAGCATAAACTGGATATGATCATTTCTGACTGTCCGA

TAGACTCTACGCAGCAGGAAGGCCTGTTCTCCGTGAGAATTGGCGAATGTGGCGTGAGTTTCTGGTGTACAAATCCACCA

CCAGAAAAACCGTTCCCGGCTTGTCTGGAAGAACGGCGACTTTTGATTCCTGGGCGACGTTCAATGTTAGGGCGCAAATT

GCTTAACTGGTTTAACTCCCAGGGATTAAACGTAGAAATCCTCGGCGAGTTTGATGATGCCGCGTTGATGAAAGCTTTTG
```

GTGCGATGCACAATGCAATCTTCGTTGCCCCAACGCTTTATGCATATGACTTTTATGCCGATAAAACTGTCGTAGAAATT

GGTCGCGTCGAGAATGTGATGGAAGAGTACCATGCTATTTTTGCTGAGCGGATGATTCAGCACCCGGCGGCCAGGGCGAA

ATCACCGGTCCGGTAACAAAATGGCAGGTCTCCCCTCCATTCTCCACGCGCCCACTTTTCCAAGTGGTTTTGGTGTACAG

TCAATAACATAAGCGGTTGGGGCCCCAAAATAGCAATGTGGATCGCACAAAGCTTTCACCGCGCCCCCCACTCCCAGAGA

TTTCTTCTTTTAAACCTCGGGAATTTAACCATTAACCTTTTGCCTCCTCTTTGAGTCGCGACAAACAAGCCTCTCCTACT

ACAGAGCGGTTTCTGTGTGTAATGTTCAACTCAGCATCTTCGCAAAGAGGCCTCCTC

>07-ecoli-4_F

CGTAAGTCATGTGACCTTGAGCAGGAATGCTGGATATCGTGAACTATCGCAAAGAATCCAATTTATTGTTTGACGTTGGC

GTGGCTGATGCACTTTCCAAACGTCTGGTCAGTAGCGTGCTGAATGCTGCAGTAGTAGAAGGCGAGCCGATTCATCTTCG

CTGCTTCGAATCCACCCACGAAATGCTGCTGGAGCAATTAAGCCAGCATAAACTGGATATGATCATTTCCGACTGTCCGA

TAGATTCTACGCAGCAGGAAGGCCTGTTCTCCGTGAGAATTGGCGAATGTGGCGTGAGTTTCTGGTGTACAAATCCACCG

CCGGAAAAAACGTTCCCGGCTTGTCTGGAAGAACGGCGACTTTTGATTCCTGGGCGACGTTCAATGTTAGGGCGCAAATT

GCTTAACTGGTTTAACTCCCAGGGATTAAACGTAGAAATCCTCGGCGAGTTTGATGATGCCGCGTTGATGAAAGCTTTTG

GTGCGATGCACAATGCAATCTTCGTTGCCCCAACGCTTTATGCATATGACTTTTATGCCGATAAAACTGTCGTAGAAATT

GGTCGCGTCGAGAATGTGATGGAAGAGTACCATGCTATTTTTGCTGAGCGGATGATTCAGCACCCGGCGGTCAGGCGAAT

CATACGTTCCAGCCAACAAAATGGCAGGTCTCTACTTCAATTCTCGACCGCCATTTTCTCACGTTTTTTCGGCGTAAAGT

CTATTCTTAGCCGTTGGGGCTACTAAATGCATGTGCATCGCACAGCTTTCACACGGCCCTCCACTGCCAGGATTTCTCTT

TAACTGGCGATTTACATTTACATTTGCTACCTGAGTCAGCATCAATCATTCTCTACAGCGGCCGTTTTCGCGTGAATGTA

CTACTGCAATTCCTTCTCAGCAAAGGTCTGCTCTCGCT

```
                      10        20        30        40        50        60        70        80
              ....|....|....|....|....|....|....|....|....|....|....|....|....|....|....|....|....|...
07-ecoli-1_F  ATGCTGGATATCGTGAACTATCGCAAAGAATCCAATTTATTGTTTGACGTTGGCGTGGCTGATGTACTTTCCAAACGTCTGGTCAGTA
07-ecoli-2_F  ..............C.-T..C.............-CC.G........T..C.....C..C....CTT.A.....G...........C.
07-ecoli-3_F  ...........T.........................................T..........C............C..........
07-ecoli-4_F  ................................................................C.......................

                      110       120       130       140       150       160       170       180
              ....|....|....|....|....|....|....|....|....|....|....|....|....|....|....|....|....|...
07-ecoli-1_F  CTGCAGTAGTAGAAGGCGAGCCGATTCATCTTCGCTGCTTCGAATCCACCCACGAAATGCTGCTGGAGCAATTAAGCCAGCATAAACT
07-ecoli-2_F  ....G..G..G.TG.A...A..C..A.....G..T........G.....G.........T....C..A..GC.C........C..G..
07-ecoli-3_F  .C.....G..............C.....................................................T...........
07-ecoli-4_F  ........................................................................................

                      210       220       230       240       250       260       270       280
              ....|....|....|....|....|....|....|....|....|....|....|....|....|....|....|....|....|...
07-ecoli-1_F  TTCCGACTGTCCGATAGATTCTACGCAGCAGGAAGGCCTGTTCTCCGTGAGAATTGGCGAATGTGGCGTGAGTTTCTGGTGTACAAAT
07-ecoli-2_F  C........C...........C...........G..G..A.....A..C.A...C........CA....C..............G...
07-ecoli-3_F  ...T..............C.....................................................................
07-ecoli-4_F  ........................................................................................

                      310       320       330       340       350       360       370       380
              ....|....|....|....|....|....|....|....|....|....|....|....|....|....|....|....|....|...
07-ecoli-1_F  AAACCGTTCCCAGCTTGTCTTGAAGAACGGCGGCTTTTGATTCCTGGGCGACGTTCAATGTTAGGGCGCAAATTACTTAACTGGTTTA
07-ecoli-2_F  ...G.......T..C..C..G.....G..A...T.GC..G.G..G..A..G..C......C.G.....T..G..GT.G..........
07-ecoli-3_F  ...........G........G...........A.........................................G.............
07-ecoli-4_F  ...A.......G........G...........A.........................................G.............

                      410       420       430       440       450       460       470       480
              ....|....|....|....|....|....|....|....|....|....|....|....|....|....|....|....|....|...
07-ecoli-1_F  TAAACGTAGAAATCCTTGGCGAGTTTGATGATGCCGCTTTGATGAAAGCTTTTGGTGCGATGCACAATGCAATCTTCGTTGCCCCAAC
07-ecoli-2_F  .GC.G..T..G.....C..T..A...........G..C..A.....G..G.....C....C....G....G..T..T..G..G..G.G
07-ecoli-3_F  ................C....................G..................................................
07-ecoli-4_F  ................C....................G..................................................

                      510       520       530       540       550       560       570       580
              ....|....|....|....|....|....|....|....|....|....|....|....|....|....|....|....|....|...
07-ecoli-1_F  TGACTTTTATGCCGATAAAACTGTCGTAGAAATTGGTCGCGTCGAGAATGTGATGGAAGAGTACCATGCTATTTTTGCTGAGCGGATG
07-ecoli-2_F  G..T............G.GT.G..AA....G.....G.....G........C........A..T..................A.A...
07-ecoli-3_F  ........................................................................................
07-ecoli-4_F  ........................................................................................

              ....|..
07-ecoli-1_F  GCGGTCC
07-ecoli-2_F  .......
07-ecoli-3_F  ....C.A
07-ecoli-4_F  ......A
```

O-nhaR-3-F: GCG TAC TTA ACG CCG CAT TG

O-nhaR-3-R: CCG GGA ACG GTT TTT CTA GT

O-nhaR-1-F : TTG TTT GAC GTT GGC GTG ACT

O-nhaR-1-R : CGG CAT CAT CAA ACT CAC CA

08-ecoli Gene Name: Isoleucine--tRNA ligase (*ileS*)

08-ecoli-F	TGACTCGCCGTATGTGCC
08-ecoli-R	TGCCCGGCAGATAAGTGC

>08-ecoli-1_F

AAGTCAACTGCGATCGAGCTGAAGTAGAGCAGAATACGGTAAGCCGGATGAGAAATTCACCGCCGCCGAGTTCCGCGCCA

AGTGCCGCGAATACGCGGCGACCCAGGTTGACGGTCAACGCAAAGACTTTATCCGTCTGGGCGTGCTGGGCGACTGGTCG

CACCCGTACCTGACCATGGACTTCAAAACTGAAGCCAATATCATCCGCGCGCTGGGCAAAATCATCGGCAATGGTCACCT

GCACAAAGGCGCGAAGCCGGTGCACTGGTGCGTAGACTGCCGTTCTGCACTGGCAGAAGCGGAAGTTGAGTATTACGACA

AAACTTCTCCGTCCATTGACGTCGCTTTCCAGGCGGTCGATCAGGATGCGCTGAAAGCGAAATTTGGCGTAAGCAACGTT

AACGGCCCAATCTCGCTGGTGATCTGGACCACTACGCCGTGGACTCTGCCTGCGAACCGCGCAATCTCTATTGCACCTGA

TTTCGACTATGCGCTGGTGCAGATCGACGGTCAGGCCGTGATTCTGGCGAAAGATCTGGTTGAAAGCGTAATGCAGCGTA

TCGGCGTGACCGATTACACCATTCTCGGCACGGTAAAAGGTGCGGAGCTTGAGCTGCTGCGCTTTGCCCATCCGTTTATG

GGCTTCGACGTCCCGGCAATCCTCGGCGATCACGTTACCCTGGATGCCGGTACCGGTGCCGTTCACACCGCGCCTGGCCA

CGGCCCGGACGACTATGTGATCGGTCAGAAATACGGCCTGGAAACCGCTGACCCGGTTGGCCCGGACGGCACTACTTTGC

CCGAGGCAAATTAACGGTTTTCGGCCTAATTTTTGACCGAATAACTTATTCTTTCGGGCCGTGCCCGGCCCGGTTGTGAA

CGGCCTCGGTTCCGGCTTCTGGGTTACGTGTATCGCCGAGTAATGGCGGAACCTCAAACTATAACGGAATTGGGCAAAGC

CAACACTAAGTTCCATCTTTTTCACTGTCTAAAAAAGGATAATGTGCCCAATTCTAATTACTTTTTTCGAAATTTGCGAA

AGACGTGCGTATATGTCCCGCAGATTTCAAATAGGTTGATATAGTTTGCTTGCCTAGCTGATCGACTAAGGGTGCCATCA

CTACAACATATGTGGGCG

>08-ecoli-2_F

AAGTCACTGCGTATCGAGCTGAAGTAGAGCAGATACGGTAAGCCGGATCGAGAAATTCACCTGCCGCCTGAGTTCCGCGC

CAAGTGCCGCGAATACGCGGCGACCCAGGTTGACGGTCAACGCAAAGACTTTATCCGTCTGGGCGTGCTGGGCGACTGGT

CGCACCCGTACCTGACCATGGACTTCAAAACTGAAGCCAATATCATCCGCGCGCTGGGCAAAATCATCGGCAATGGTCAC

CTGCACAAAGGCGCGAAGCCGGTGCACTGGTGCGTAGACTGCCGTTCTGCACTGGCAGAAGCGGAAGTTGAGTATTACGA

CAAAACTTCTCCGTCCATTGACGTCGCTTTCCAGGCGGTCGATCAGGATGCGCTGAAAGCGAAATTTGGCGTAAGCAACG

TTAACGGCCCAATCTCGCTGGTGATCTGGACCACTACGCCGTGGACTCTGCCTGCGAACCGCGCAATCTCTATTGCACCT

GATTTCGACTATGCGCTGGTGCAGATCGACGGTCAGGCCGTGATTCTGGCGAAAGATCTGGTTGAAAGCGTAATGCAGCG

TATCGGCGTGACCGATTACACCATTCTCGGCACGGTAAAAGGTGCGGAGCTTGAGCTGCTGCGCTTTGCCCATCCGTTTA

TGGGCTTCGACGTCCCGGCAATCCTCGGCGATCACGTTACCCTGGATGCCGGTACCGGTGCCGTTCACACCGCGCCTGGC

CACGGCCCGGACGACTATGTGATCGGTCAGAAATACGGCCTGGAAACCGCTAACCCGGTTGGCCCGGACGGCACTTACTT

GGCCCGGAGCAAGTAAGGCGGTTCTCGGCCGTAATTTCTGAACCGATCACATAGTCTTCCGGGCCGTGCGCGGGCGCGGT

GTGAAACGGGCCCGGTTCGGGCTTCGGGGTTACGTGATTCGCTAGATGGCCGGGACTTCTACATAGCGTAGTTGGCGAGA

GCAACACTCGTCTGCCTTACGTGCAAAGGGGTTAATGCTAGCCATTCTTGTTACTTTTCGAATTTTGCGATACAGTGCAC

TGT

>08-ecoli-3_F

TAGGTCAATGCGATCGAGCTGAAGTAGAGCAAGAATACGGTAAGCCGGGTGAGAAATTCACCGCCGCCGAGTTCCGCGCC

AAGTGCCGCGAATACGCGGCGACCCAGGTTGACGGTCAACGCAAAGACTTTATCCGTCTGGGCGTGCTGGGCGACTGGTC

GCACCCGTACCTGACCATGGACTTCAAAACTGAAGCCAACATCATCCGCGCGCTGGGCAAAATCATCGGCAACGGTCACC

TGCACAAAGGCGCGAAGCCAGTTCACTGGTGCGTTGACTGCCGTTCTGCGCTGGCGGAAGCGGAAGTTGAGTATTACGAC

AAAACTTCTCCGTCCATCGACGTTGCTTTTCAGGCGGTCGATCAGGATGCACTGAAAGCAAAATTTGCCGTAAGCAACGT

TAACGGCCCAATCTCGCTGGTGATCTGGACCACTACGCCGTGGACTCTGCCTGCGAACCGCGCAATCTCTATTGCACCTG

ATTTCGACTATGCGCTGGTGCAGATCGACGGTCAGGCCGTGATTCTGGCGAAAGATCTGGTTGAAAGCGTAATGCAGCGT

ATCGGCGTGACCGATTACACCATTCTCGGCACGGTAAAAGGTGCGGAGCTTGAGTTGCTGCGCTTTACCCATCCGTTTAT

GGGCTTCGACGTTCCGGCAATCCTCGGCGATCACGTTACCCTGGATGCCGGTACCGGTGCCGTTCACACCGCGCCTGGCC

ACGGCCCGGACGACTATGTGATCGGTCAGAAAATACGGCCTGGAAACCGCTAACCCGGTTGGCCCGGACGGCACTACTTG

GGCCGGGGCACGGGTTTAGCGGTTTCAGGGCGTATTTTCTGACCGAATAACATAGTCTTCCGGGGCCGTGGCCGGGCCGG

GTGTTAAACGGCCCCGGTTCCGGCCTCTCGGGGTTACGTGATCGCGCAGAGATTTGCCGAACGTTCAAACATAACGGATG

GGTTAGGGCACACTTCAGTCGCACTCTTACGTGCCGAGACAGGGGTATGGGTACGCATCCTCACTACTTCAGATTTTGCG

AATCGCGTACGGTGATGCACAGGAAGTCAATAGGGCTTAAAATTGCGTGCCAGGCAAGACAGCCAAGGTCAATCAACGAA

ATGGTGCTGACGTTTAAGTTAAGTGCTTCTGATGCTA

>08-ecoli-4_F

AGTCAATGCGATCGAGCTGAAGTAGAGCAAGAATACGGTAAGCCGGGTGAGAAATTCACCGCCGCCGAGTTCCGCGCCAA

GTGCCGCGAATACGCGGCGACCCAGGTTGACGGTCAACGCAAAGACTTTATCCGTCTGGGCGTGCTGGGCGATTGGTCGC

ACCCGTACCTGACCATGGACTTCAAAACTGAAGCCAATATCATCCGCGCGCTGGGCAAAATCATCGGCAATGGTCACCTG

CACAAAGGCGCGAAGCCGGTGCACTGGTGCGTAGACTGCCGTTCGGCACTGGCAGAAGCGGAAGTTGAGTATTACGACAA

AACTTCTCCGTCCATTGACGTCGCTTTCCAGGCGGTCGATCAGGATGCGCTGAAAGCGAAATTTGGCGTAAGCAACGTTA

ACGGCCCAATCTCGCTGGTGATCTGGACCACTACGCCGTGGACTCTGCCTGCGAACCGCGCAATCTCTATTGCACCTGAT

TTCGACTATGCGCTGGTGCAGATCGACGGTCAGGCCGTGATTCTGGCGAAAGATCTGGTTGAAAGCGTAATGCAGCGTAT

CGGCGTGACCGATTACACCATTCTCGGCACGGTAAAAGGTGCAGAGCTTGAGCTGCTGCGCTTTACCCATCCGTTTATGG

GCTTCGACGTTCCGGCAATCCTCGGCGATCACGTTACCCTGGATGCCGGTACCGGTGCCGTTCACACCGCGCCTGGCCAC

GGCCCGGACGACTATGTGATCGGTCAGAAATACGGCCTGGAAACCGCTAACCCGGTTGGCCCGGACGGCACTATCTTTGC

CCGAGAGCAGGTTAACGGGTTTCGGCCGTATTTCTTGAACAGATACATTAGTTCTTCGGGCCGGGGCCGGGCCGCGGTGT

GTGTACGGCCCGGTCCGCCTTCGGGTACGTGTTCGCAGGATTTGCGGACGGTCATCATTACGGTATGGGTAAGGCAACAC

CTAGCTCTCCTTTCTTCGTGTGCGAGCAAGGGGTGTATTGCGTAG

```
                      10        20        30        40        50        60        70        80
              ....|....|....|....|....|....|....|....|....|....|....|....|....|....|....|....|....|...
08-ecoli-1_F  -AAGTCAACTGCG-ATCGAGCTGAAGTAGAGCA-GAATACGGTAAGCCGGAT-GAGAAATTCACC-GCCGCC-GAGTTCCGCGCCAAG
08-ecoli-2_F  -......-.....T...................-..-...............C............T......T...............
08-ecoli-3_F  T.G....-A....-...................A................G.-............-......-...............
08-ecoli-4_F  --.....-A....-...................A................G.-............-......-...............

                     110       120       130       140       150       160       170       180
              ....|....|....|....|....|....|....|....|....|....|....|....|....|....|....|....|....|...
08-ecoli-1_F  GCGGCGACCCAGGTTGACGGTCAACGCAAAGACTTTATCCGTCTGGGCGTGCTGGGCGACTGGTCGCACCCGTACCTGACCATGGACT
08-ecoli-2_F  ........................................................................................
08-ecoli-3_F  ........................................................................................
08-ecoli-4_F  ...........................................................T............................

                     210       220       230       240       250       260       270       280
              ....|....|....|....|....|....|....|....|....|....|....|....|....|....|....|....|....|...
08-ecoli-1_F  CCAATATCATCCGCGCGCTGGGCAAAATCATCGGCAATGGTCACCTGCACAAAGGCGCGAAGCCGGTGCACTGGTGCGTAGACTGCCG
08-ecoli-2_F  ........................................................................................
08-ecoli-3_F  ....C................................C..........................A..T...........T........
08-ecoli-4_F  ........................................................................................

                     310       320       330       340       350       360       370       380
              ....|....|....|....|....|....|....|....|....|....|....|....|....|....|....|....|....|...
08-ecoli-1_F  AGAAGCGGAAGTTGAGTATTACGACAAAACTTCTCCGTCCATTGACGTCGCTTTCCAGGCGGTCGATCAGGATGCGCTGAAAGCGAAA
08-ecoli-2_F  ........................................................................................
08-ecoli-3_F  G.........................................C.....T.....T....................A........A...
08-ecoli-4_F  ........................................................................................

                     410       420       430       440       450       460       470       480
              ....|....|....|....|....|....|....|....|....|....|....|....|....|....|....|....|....|...
08-ecoli-1_F  AACGTTAACGGCCCAATCTCGCTGGTGATCTGGACCACTACGCCGTGGACTCTGCCTGCGAACCGCGCAATCTCTATTGCACCTGATT
08-ecoli-2_F  ........................................................................................
08-ecoli-3_F  ........................................................................................
08-ecoli-4_F  ........................................................................................

                     510       520       530       540       550       560       570       580
              ....|....|....|....|....|....|....|....|....|....|....|....|....|....|....|....|....|...
08-ecoli-1_F  TGGTGCAGATCGACGGTCAGGCCGTGATTCTGGCGAAAGATCTGGTTGAAAGCGTAATGCAGCGTATCGGCGTGACCGATTACACCAT
08-ecoli-2_F  ........................................................................................
08-ecoli-3_F  ........................................................................................
08-ecoli-4_F  ........................................................................................

                     610       620       630       640       650       660       670       680
              ....|....|....|....|....|....|....|....|....|....|....|....|....|....|....|....|....|...
08-ecoli-1_F  AAAAGGTGCGGAGCTTGAGCTGCTGCGCTTTGCCCATCCGTTTATGGGCTTCGACGTCCCGGCAATCCTCGGCGATCACGTTACCCTG
08-ecoli-2_F  ........................................................................................
08-ecoli-3_F  ...................T...........A.........................T..............................
08-ecoli-4_F  .........A.....................A.........................T..............................

                     710       720       730       740       750       760       770       780
              ....|....|....|....|....|....|....|....|....|....|....|....|....|....|....|....|....|...
08-ecoli-1_F  GGTGCCGTTCACACCGCGCCTGGCCACGGCCCGGACGACTATGTGATCGGTCAGAAA-TACGGCCTGGAAACCGCTGACCCGGTTGGC
08-ecoli-2_F  .........................................................-..................A...........
08-ecoli-3_F  .........................................................A..................A...........
08-ecoli-4_F  .........................................................-..................A...........
```

ileS1-3 F : ACA AAG GCG CGA AGC CAA TT

ileS1-3 R : GGA TGG GTA AAG CGC AGT AA

ileS1-4 F : TCT GGG CGT GCT GGG CAA T

ileS1-4 R : AGC GCA GCA GCT CAA GTT CT

09-ecoli marker, Gene Name: Isoleucine--tRNA ligase (*ileS*)

09-ecoli-F	CGGCCTGGAAACCGCTAA
09-ecoli-R	TCGGTTGATGCCACCCAC

>09-ecoli-1_F

GTGGGTTCGCTTATCTGCGGGCACTTATCCGACGCTGGATGGCGTGAACGTCTTCAAAGCGAACGACATCGTCGTTGCGC

TGCTGCAGGAAAAAGGCGCGCTGCTGCACGTTGAGAAAATGCAGCACAGCTATCCGTGCTGCTGGCGTCACAAAACGCCG

ATCATCTTCCGCGCAACGCCGCAGTGGTTCGTCAGTATGGATCAGAAAGGTCTGCGCGCGCAGTCTCTGAAAGAGATCAA

AGGTGTGCAGTGGATCCCGGACTGGGGCCAGGCGCGTATCGAGTCGATGGTCGCTAACCGTCCTGACTGGTGTATCTCCC

GTCAGCGTACCTGGGGCGTACCGATGTCTCTGTTCGTGCACAAAGACACGGAAGAGCTGCATCCGCGTACCCTCGAACTG

ATGGAAGAAGTGGCTAAACGCGTTGAAGTTGATGGCATCCAGGCGTGGTGGGATCTTGATGCGAAAGAGATCCTCGGCGA

TGAAGCTGATCAGTATGTGAAAGTGCCGGATACGCTGGATGTATGGTTTGACTCCGGCTCTACTCACTCTTCTGTTGTTG

ACGTGCGCCCGGAATTTGCCGGTCACGCTGCGGACATGTATCTGGAAGGTTCTGACCAACACCGTGGCTGGTTCATGTCT

TCTCTGATGATCTCCACCGCGATGAAGGGCAAAGCGCCGTATCGTCAGGTGCTGACCCACGGCTTTACCGTAGATGGTCA

GGGCCGCAAGATGTCTAAATCCATCGGCAACACCGTGTCGCCGCAGGATGTGATGAACAAACTGGGCGCGGATATTCTGC

GTCTGTGGGTGCATTTCAACCCGAAAAAAAAGAACTACGATTCCTTCTGTTTTTTTGGCTGGTCTTTGCCGTTGTTCTCA

CGTTTTCTTCTTAGTCTTTTCACTTCTTGTGGCGTTTAGGTCCTGTCGTTTCTGTCTTGCGGG

>09-ecoli-2_F

GTAGGCTCACTACTGCAGGCACATATCCACGCTTGACGGTGTTGACGGCTTCAGGCGAACGATATCGTTATTGAACTGCT

GAAAGAAAAAGGCGCGCTACTGCATGTCGAAAAAATGGAGCACAGCTATCCGTGCTGTTGGCGTCACAAAACGCCGATCA

TTTTCCGCGCGACCCCGCAGTGGTTCGTCAGCATGGATAAAGAAGGGCTTCGTCAACAGTCCCTGAAAGAGATCAAAGGC

GTTCAGTGGATCCCGGACTGGGGTCAGGCGCGTATTGAATCAATGGTCGCTAACCGTCCTGACTGGTGTATCTCACGTCA

GCGTACCTGGGGCGTACCGATGTCGCTGTTCGTGCATAAAGAAACGCAGGAGCTGCTGCCGATTGACCGTACTCTGGCGG

CGATGGAAGAGGTGGCGAAACGCGTTGAAGTTGACGGTATCCAGGGGTGGTGGGATCTCGACCCGAAAGAGATCCTCGGC

GAAGATGCCGATCAATACGAGAAAGTTCCGGATACGTTGGACGTTTGGTTTGATTCCGGTTCCACCAGTTACTCCGTGGT

TGATGCACGTCCGGAATTCGCCGGTCATGCTGCCGATATGTATCTGGAAGGGTCTGACCAGCATCGCGGCTGGTTTATGT

CCTCCCTGATGATTAGCGTGGCGATGAAAGGCAAAGCGCCATACCGCCTGGTACTGACTCACGGCTTCACCGTCGATGGT

CAGGGGCGCAAGATGTCCAAGTCCATCGGTAACACCGTGTCGCCACAGGATGTGATGAATAAACTGGGCGCGGCATATCC

TGCGTCTGTGGGTGGCATCAACCCGAAAGAAAGGTAACTCGCACCTCCTGTTCCTCTTTTGCAGTGTGCTTGCGATCTCC

AGGTTAAAGATTTCCTGTTTTGTACCTGCTGACTCATTAGGTCCGCTTATGCATGTCTAAAAAAATTTAGCGATCTTTGT

CTCGTCTGTTTTGCTTATCTACCCTTTATTATTTTTTTCGTTCTCTCCTTCTTG

>09-ecoli-3_F

ATGTGCTACCGCATATCTGCGGGCACTTATCCGACGCTGGATGGCGTGAACGTCTTCAAAGCGAACGACATCGTCGTTGC

GCTGCTGCAGGAAAAAGGCGCGCTGCTGCACGTTGAGAAAATGCAGCACAGCTATCCGTGCTGCTGGCGTCACAAAACGC

CGATCATCTTCCGCGCGACGCCGCAGTGGTTCGTCAGCATGGATCAGAAAGGTCTGCGTGCGCAGTCACTGAAAGAGATC

AAAGGCGTGCAGTGGATCCCGGACTGGGGCCAGGCGCGTATCGAGTCGATGGTTGCTAACCGTCCTGACTGGTGTATCTC

CCGTCAGCGCACCTGGGGCGTACCGATGTCACTGTTCGTGCACAAAGACACGGAAGAGCTGCATCCGCGTACTCTCGAAC

TAATGGAAGAAGTGGCAAAACGCGTTGAAGTTGACGGCATCCAGGCGTGGTGGGATCTTGATGCGAAAGAGATCCTCGGC

GATGAAGCTGATCAGTACGTGAAAGTGCCGGACACATTGGATGTATGGTTTGACTCCGGATCTACCCACTCTTCTGTTGT

TGACGTGCGTCCGGAATTTGCCGGTCACGCAGCGGACATGTATCTGGAAGGTTCTGACCAGCACCGTGGTTGGTTCATGT

CTTCCCTGATGATCTCCACCGCGATGAAAGGCAAAGCGCCGTATCGTCAGGTACTGACCCACGGCTTTACCGTGGATGGT

CAGGGTCGCAAGATGTCTAAATCCATCGGCAACACCGTTTCGCCGCAGGATGTGATGAACAAACTGGGGCGCGGATATTC

TGCGTCTGTGGGTGCAATTCCAACCCGAAAAAAGGAATTTCAGGCCCCTCGTCTGTTTTTTAATTGCCGGCGAGGAAATC

AATGTTTACCGATATAGTCTTTTTACTTCTTGTCTCGCGAAACAAGCCACGTTGTCTGTACGTGTGTGGAATATGCATAC

CAAATCTGTCCCTCTTGCTGTCTCGCCCGAGGGCAGATATAACCTGGGTAGAAAACTATCGACCCCCGCCGTTCTGTTGT

AAACTTCTCTATACTATGCCTCCGTGCTGCGCTAATTATGATTCATCTGATGCATA

>09-ecoli-4_F

GAGCCGCCGCTTATCTGCGGGCACTTATCCGAACGCTGGTAAGGGCGTGAACGTCTTCAAAGCGAACGACATCGTCGTGG

CGCTCGCTCGCAGGAAAAAGGCGCTCTGCTGCACGTTGAGAAAATGCAGCACAGCTATCCGTGCTGCTGGCGTCACAAAA

CGCCGATCATCTTCCGCGCGACACCGCAGTGGTTCGTCAGCATGGATCAGAAAGGTCTGCGTGCGCAGTCACTGAAAGAG

ATCAAAGGCGTGCAGTGGATCCCGGACTGGGGCCAGGCGCGTATCGAGTCGATGGTTGCTAACCGTCCTGACTGGTGTAT

CTCCCGTCAGCGCACCTGGGGCGTACCGATGTCACTGTTCGTGCACAAAGACACGGAAGAGCTGCATCCGCGTACCCTCG

AACTAATGGAAGAAGTGGCAAAACGCGTTGAAGTTGACGGCATCCAGGCGTGGTGGGATCTTGATGCGAAAGAGATCCTC

GGCGATGAAGCTGATCAGTACGTGAAAGTGCCGGACACATTGGATGTATGGTTTGACTCCGGATCTACCCACTCTTCTGT

TGTTGACGTGCGTCCGGAATTTGCCGGTCACGCAGCGGACATGTATCTGGAAGGTTCTGACCAGCACCGCGGTTGGTTCA

TGTCTTCCCTAATGATCTCCACCGCGATGAAGGGCAAAGCGCCGTATCGTCAGGTACTGACCCACGGCTTTACCGTGGAT

GGTCAGGGTCGCAAGATGTCTAAATCCATCGGCAACACCGTTTCGCCGCAGGATGTGATGAACAAACTGGGTGCGGATAT

TCTGCGTCTGTGGGTGCATATCCAACCCGAAAAAAAGGAGGGCTTTCCATCCCTGTTGGTTCTTATATGCCGCGGCGAAT

CGTTGGTTGCTGACAGATGTATAGTCTCTGTCTGCCGCACCATCCCCCGCTGTAAGCACTGTGTGAATATCTGAACTAGC

TCTGTCCTGTTGCTTTCCTATCGCAGTGAAATATATTTCTGGGCATGACTATATCATCTTCGGTGCTTGTCTATAAAGAT

ACTCTTATAGTATTTCTCTCTCGCGTGGGTGCGATATGTACTA

```
                       10        20        30        40        50        60        70        80
              ....|....|....|....|....|....|....|....|....|....|....|....|....|....|....|....|....|...
09-ecoli-1_F  CTTATCCGA-CGCTGG--ATGGCGTGAACGTCTTCAAAGCGAACGACATCGTCGTTGCGCT-GCT-GCAGGAAAAAGGCGCGCTGCTG
09-ecoli-2_F  .A.....A--....T.--.C..T..TG...G.....G-........T.....TA...AA..-...-.A.A..............A...
09-ecoli-3_F  .........-......--...........................................-...-......................
09-ecoli-4_F  .........A......TA.G...................................G.....C...C...............T......

                      110       120       130       140       150       160       170       180
              ....|....|....|....|....|....|....|....|....|....|....|....|....|....|....|....|....|...
09-ecoli-1_F  ATGCAGCACAGCTATCCGTGCTGCTGGCGTCACAAAACGCCGATCATCTTCCGCGCAACGCCGCAGTGGTTCGTCAGTATGGATCAGA
09-ecoli-2_F  ...G...................T.......................T........G..C.................C......A.AG
09-ecoli-3_F  ........................................................G....................C..........
09-ecoli-4_F  ........................................................G..A.................C..........

                      210       220       230       240       250       260       270       280
              ....|....|....|....|....|....|....|....|....|....|....|....|....|....|....|....|....|...
09-ecoli-1_F  CGCAGTCTCTGAAAGAGATCAAAGGTGTGCAGTGGATCCCGGACTGGGGCCAGGCGCGTATCGAGTCGATGGTCGCTAACCGTCCTGA
09-ecoli-2_F  AA.....C.................C..T....................T...........T..A..A....................
09-ecoli-3_F  .......A.................C...............................................T..............
09-ecoli-4_F  .......A.................C...............................................T..............

                      310       320       330       340       350       360       370       380
              ....|....|....|....|....|....|....|....|....|....|....|....|....|....|....|....|....|...
09-ecoli-1_F  CCGTCAGCGTACCTGGGGCGTACCGATGTCTCTGTTCGTGCACAAAGACACGGAAGAGCTGCATCCG------CGTACCCTCGAACTGA
09-ecoli-2_F  A.............................G...........T.....A...C.G.......TG...ATTGAC.....T..G.CGGC.
09-ecoli-3_F  .........C....................A....................................------.....T........A
09-ecoli-4_F  .........C....................A....................................------..............A

                      410       420       430       440       450       460       470       480
              ....|....|....|....|....|....|....|....|....|....|....|....|....|....|....|....|....|...
09-ecoli-1_F  GCTAAACGCGTTGAAGTTGATGGCATCCAGGCGTGGTGGGATCTTGATGCGAAAGAGATCCTCGGCGATGAAGCTGATCAGTATGTGA
09-ecoli-2_F  ..G.................C..T.......G............C..CC...................A..T..C.....A..C.A..
09-ecoli-3_F  ..A.................C..............................................................C....
09-ecoli-4_F  ..A.................C..............................................................C....

                      510       520       530       540       550       560       570       580
              ....|....|....|....|....|....|....|....|....|....|....|....|....|....|....|....|....|...
09-ecoli-1_F  CGCTGGATGTATGGTTTGACTCCGGCTCTACTCACTCTTCTGTTGTTGACGTGCGCCCGGAATTTGCCGGTCACGCTGCGGACATGTA
09-ecoli-2_F  ..T....C..T........T.....T..C..CAGT.AC..C..G.....T.CA..T........C........T.....C..T.....
09-ecoli-3_F  .AT......................A.....C.......................T....................A...........
09-ecoli-4_F  .AT......................A.....C.......................T....................A...........

                      610       620       630       640       650       660       670       680
              ....|....|....|....|....|....|....|....|....|....|....|....|....|....|....|....|....|...
09-ecoli-1_F  TGACCAACACCGTGGCTGGTTCATGTCTTCTCTGATGATCTCCACCGCGATGAAGGGCAAAGCGCCGTATCGTCAGGTGCTGACCCAC
09-ecoli-2_F  ......G..T..C........T.....C..C........TAG.GTG........A...........A..C..C.T...A.....T...
09-ecoli-3_F  ......G........T..............C.......................A.......................A.........
09-ecoli-4_F  ......G.....C..T..............C..A............................................A.........

                      710       720       730       740       750       760       770       780
              ....|....|....|....|....|....|....|....|....|....|....|....|....|....|....|....|....|...
09-ecoli-1_F  GATGGTCAGGGCCGCAAGATGTCTAAATCCATCGGCAACACCGTGTCGCCGCAGGATGTGATGAACAAACTGGG-CGCGG-ATATTCT
09-ecoli-2_F  ...........G...........C..G........T..............A..............T........-.....C....C..
09-ecoli-3_F  ...........T................................T.............................G.....-.......
09-ecoli-4_F  ...........T................................T.............................-T....-.......

                      810
              ....|....|...
09-ecoli-1_F  GCAT-TTCAACCC
09-ecoli-2_F  .GCA--.......
09-ecoli-3_F  ...AT.C......
09-ecoli-4_F  ....A.C......
```

ileS2-1 F : GAT CAT CTT CCG CGC AGC G

ileS2-1 R : CAA CAA CAG AAG AGT GAG TAT AG

ileS2-3 F : CGA TCA TCT TCC GCG CGC CG

ileS2-3 R : GAG TCA AAC CAT ACA TCC AAT TTG

14-ecoli, Gene Name: Carbamoyl-phosphate synthetase subunit beta (*carB*)

14- ecoli-F	TTGCTAAAGTGGCGGCGA
14- ecoli-R	AGACGGATTCGCTTCGCA

>14-ecoli-1_F

GAATCGGTAACCTCGATGACTGATGACGACATCACTGGCGGACGTACTCCGGCCTCCTTCGAGCCGTCCATCGATTACGT

GGTTACCAAAATTCCTCGCTTCAACTTCGAAAAATTCGCCGGTGCTAACGACCGTCTGACCACTCAGATGAAATCGGTTG

GCGAAGTGATGGCGATTGGTCGCACGCAGCAGGAATCCCTGCAAAAAGCGCTGCGCGGCCTGGAAGTCGGTGCGACTGGA

TTCGACCCGAAAGTGAGCCTGGATGACCCGGAAGCGTTAACCAAAATCCGTCGCGAACTGAAAGACGCAGGCGCAGAGCG

TATCTGGTACATCGCCGATGCTTTCCGCGCGGGCCTGTCTGTGGACGGCGTCTTCAACCTGACTAACATTGACCGCTGGT

TCCTGGTACAGATTGAAGAACTGGTGCGTCTGGAAGAGAAAGTGGCGGAAGTGGGCATCACTGGCCTGAACGCTGAATTC

CTGCGCCAGCTGAAACGCAAAGGCTTTGCCGATGCGCGCTTGGCAAAACTGGCGGGCGTACGCGAAGCGGAAATCCGTAA

GCTGCGTGACCAATATGACCTGCACCCGGTCTACAAGCGCGTGGATACCTGTGCGGCAGAGTTTGCCACCGACACCGCTT

ACATGTACTCCACTTATGAAGAAGAGTGCGAAGCGAAATCCGTCTA

>14-ecoli-2_F

AGCGGTCCGGAATTCTCTAATACCGAATTCAGACTCACGGTGGGAAGGTGTGCAGGCCTCTTAGAGGAAGACCTTCGTTA

AGGCGGTTACAAAAGTTTCCTGTGTGAAGGTGAAAGAATTCACCGTGGCTAACGACCGCCCGCCCTCTTGGTGGAAATCG

GTTGGCGAATGAAGGCGAAACGCCGCAGGCACAAGGAAGCCTGGCAAAAAGGGCTGTGCGGCCTGTAACTCGGTGCGACT

GAATCCGACCGGAAGGTTTCCCGGGAGGACCCGGAAACGTTAACCAAATTCCTACCCAATCCGAATGTGACGGAAAAATC

GGCATCCGGTCCTTCACCAAAGTTTTGTTAGCGGATCTGCCGGTTGACAGCGACTTATACTTGACTAGTTTTGACGCCCG

GTAACTGAAACTGATTGAACAATTGTTTCTTCTCTGCTTATATAGTGTTATTTTTTTTAATTTTGATTTTATCTTTCCAC

TATCTTTCTCATTGTAAATTCATAATTGATAAGTAATTAATCATTCTCAATACTCCTGCGAGGCCCAATCTGTTTACGCT

TTTGTTATCTTACAATTAAGAGTTGTTAACGTTTTATTATTGTTAGTTTTATTTTTTCTCTATATTCGACTCCTATTATC

TTCTTTTAATTTCTCATATTTTAATTCATTTTTTAATTTTTGTTTTTTTTTTTTTTTATTCTCTTTTTTTCTTTTCTTCT

TTTCTATTCTTTTTGTTTGTTTTCTTTTTTTTCTGATTTGTCTATATCTTTATTTTTTTCTCTGTAGTTTTTATCTTTTT

ATATTGG

>14-ecoli-3_F

GAATCGTTAACTCGAGACTGATGACGACATCACTGGCGGACGTACTCCGGCCTCCTTCGAGCCGTCCATCGACTACGTGG

TTACCAAAATTCCTCGCTTCAACTTCGAAAAATTCGCCGGTGCTAACGACCGTCTGACCACTCAGATGAAATCGGTTGGC

GAAGTGATGGCGATTGGTCGCACGCAGCAGGAATCCCTGCAAAAAGCGCTGCGCGGCCTGGAAGTCGGTGCGACTGGATT

CGACCCGAAAGTGAGCCTGGATGACCCGGAAGCGTTAACCAAAATCCGTCGCGAACTGAAAGACGCTGGCGCAGAGCGTA

TCTGGTACATCGCCGATGCTTTCCGCGCGGGCCTGTCTGTGGACGGCGTCTTCAACCTGACTAACATTGACCGCTGGTTC

CTGGTACAGATTGAAGAACTGGTGCGTCTGGAAGAGAAAGTGGCGGAAGTGGGCATCACTGGCCTGAACGCTGAATTCCT

GCGCCAGCTGAAACGCAAAGGCTTTGCCGATGCGCGCTTGGCAAAACTGGCGGGCGTACGCGAAGCGGAAATCCGTAAGC

TGCGTGACCAATATGACCTGCACCCGGTCTATAAGCGCGTGGATACCTGTGCGGCAGAGTTCGCCACCGACACCGCTTAC

ATGTACTCCACTTATGAAGAAGAGTGCGAAGGAAAATCCGTCTA

>14-ecoli-4_F

TGTCGTGTTCTTCATCTTTTACTAAAAATTAACTTCACTGTGGATGGAGCTCCGGTCTCCTTCCAGAGACCGTCGTCGAC

GAGGGATAAATTAAGGAGGAAATGGGAGAATCCCGGTGGTAACGACCGTCTGATTACAAGAGGAAATCGTTGTCGAATGA

TGTTGGGGGGTCGCACGCATCAAAAATTTTTGCATATACTTTTGCGCGGGCTGAAAGTCTATATATACTGTTATTCGACC

CGAAAGGGATCCTCGATGACCCGGAAGCGTTAACCTAAATCCGGTGCAAACTGAAAGAAGAGAGTATATAGAGTATCTAG

TACTTCTTCTACGCTTTCTGTGTGGGTCTGTCTGCGGACGAAGACTACTACCTGACTCCGATTGACCGCTATTTGATGAT

ACCGATTTAATAACTGTGTTGTCTTTCCTATAAAGAAGATTCTTCGGCAATCATTTGTTATTACTTCTTAAATCCTTTTT

TTATCTTAATGGTATGTGTTTTATGATTGAT

```
                      10        20        30        40        50        60        70        80
              ....|....|....|....|....|....|....|....|....|....|....|....|....|....|....|....|....|...
14-ecoli-1_F  GGTAACCTCGATGACTGATGAC-GACATCACTG-GCGGACGTA-CTCCGGCCTCCTTCGAGCCGTCCATCGATTACGTGGTTACCAAA
14-ecoli-3_F  .T....-....-..........-..........-.........-............................C...............
14-ecoli-2_F  ..A.TT...T.AT..C..ATT.A...-....G.T.G.A.G..G-TG.A......T.AGAG.AA.A..T...T.A.G.C.....A...
14-ecoli-4_F  CT.C.T..TTT--...A.AA.TTA..T......--T...T.G.G......T.......C..A-.A..G...TCG...A..--GAT...

                     110       120       130       140       150       160       170       180
              ....|....|....|....|....|....|....|....|....|....|....|....|....|....|....|....|....|...
14-ecoli-1_F  AACTTCGAAAAATTCGCCGGTGCTAACGACCGTCTGACCACTCAGATGAAATCGGTTGGCGAAGTGATGGCGATTGGTCGCACGCAGC
14-ecoli-3_F  ........................................................................................
14-ecoli-2_F  ..GG.GA..G.....A...TG............C.C.C..T..TG.TG...................-...A.....AAC.C....G...CA
14-ecoli-4_F  G.AA.G.G.G...C.--.....G...............TT..A-...G.......-...T....-.....TT.GGG..........T.

                     210       220       230       240       250       260       270       280
              ....|....|....|....|....|....|....|....|....|....|....|....|....|....|....|....|....|...
14-ecoli-1_F  AAAAAGCGCTGCGCGGCCTGGAAGTCGGTGCG-ACTGG-ATTCGACCCGAAAGTGAGCCTGGATGACCCGGAAGCGTTAACCAAAATC
14-ecoli-3_F  ................................-.....-.................................................
14-ecoli-2_F  ......G....T........T..C........-....A-..C.....G...G..TTC..G...G.........A...........T..
14-ecoli-4_F  .T.T.CTTT.......G...A.....TA.ATAT....TT..............G..T...C......................T.....

                     310       320       330       340       350       360       370       380
              ....|....|....|....|....|....|....|....|....|....|....|....|....|....|....|....|....|...
14-ecoli-1_F  AAAGACGCAGGCGCAGAGCGTATCTGGTACATCGCCGATGCTTTCCGCGCGGGCCTGTCTGTGGACGGCGTCTTCAACCTGACTAACA
14-ecoli-3_F  ........T...............................................................................
14-ecoli-2_F  ..T.TGA.G.AAAA.TC.-.C...C...C.T..A..A.A.T...GTTA....AT...C.G..T...A...A...AT..T......GTT
14-ecoli-4_F  .....A.AGA.TAT.T..A.......A....T..TT.T.C......T.T.T...T.......C.....AA.A..A.T........CCG.

                     410       420       430       440       450       460       470       480
              ....|....|....|....|....|....|....|....|....|....|....|....|....|....|....|....|....|...
14-ecoli-1_F  TCCTGGTACAGATTGAAGAACTGGTGCGTCT-GGAAGAGAAAGTGGCGGAAGTGGGCATCACTGGCCTGAACGC--TGAATTCCTGCG
14-ecoli-3_F  ...............................-..........................................--............
14-ecoli-2_F  AA...AA..T.......C..T..T.T.T...CT.CTT.T.T....TTATTTT.TTTA..--T.T.AT.TT.TCTTTCC.C.AT..TTC
14-ecoli-4_F  .GA..A...C....T..T.....TGTT....-TTCCT.T....AA.ATTCTTC..CA....T.T.TTATT..TTCT.A...C.T.TTT

                     510       520       530       540       550       560       570       580
              ....|....|....|....|....|....|....|....|....|....|....|....|....|....|....|....|....|...
14-ecoli-1_F  CAAAGGCTTTGCC-GATGCGCGCTTGGCAAAACTGGCGGGCGTACGCGAAGCGGAAATCCGTAAGCTGCGTGACCAATATGACCTGCA
14-ecoli-3_F  .............-..........................................................................
14-ecoli-2_F  ..T.ATTGA.AAGTA..TAATCA..CT...T...CCT.C.A.-G.C.A.TCT.TTT.CG.T.TT.T.ATC.T..A.T..A..GT..TT
14-ecoli-4_F  T.TGT.T...AT--...TGAT-------------------------------------------------------------------

                     610       620       630       640       650       660       670       680
              ....|....|....|....|....|....|....|....|....|....|....|....|....|....|....|....|....|...
14-ecoli-1_F  GCGCGTGGAT-ACCTGTGCGGCAGAGTTTGCCACCGACACCGCTTACATGTACTCCACTTATGAAGAAGAGTGCGAAGCGAAATCCGT
14-ecoli-3_F  ..........-.................C.................................................GA........
14-ecoli-2_F  TT.TTA.TT.T.TT.T.T.TCT.T.T.CGA.TC.TATT.T.TTC.TTTAA.TTCT..TA.T.T..TTCATT.TTT..TTTTTG.TTT.
14-ecoli-4_F  ----------------------------------------------------------------------------------------
```

15-ecoli marker, Gene Name: Carnitine-CoA ligase (*caiC*)

15-ecoli-F	TATGCAGCCAGCCATCGG
15- ecoli-R	AGATGTGGCACTTCCCGC

>15-ecoli-1_F

CTTGGACGGGAGTGCTCACTGACTATCAACAACCTGCCACCTTGTGCTATGCACCGCCGCTATCGACTGACGATACGGAG

GATGTATCTTCACCTCCGGCACCACCTCCCGACCGAAAGGTGTGGTGATTACCCATTACAACCTGCACTTCGCTGGATAT

TACTCCGCCTGGGAGTGTGCACTGCGTGACGATGACGTCTACCTGACGATAATGCCTGCGTTTCATATCGATTGCCAGTG

TACTGCGGCGATGGCGGCGTTTTCTGCCGGGGCCACCTTTGTGCTGGTCGAGAAATACAGCGCCCGCGCCTTCTGGGGAC

AGGTACAGAAGTACCGCGCCACCATTACCGAATGTATTCCGATGATGATTCGTACGTTGATGATGCAGCCGCCTTCAGCG

AACGATCGGCAACACCGCCTGCGGGAAGTGATGTTTAATCTCAACTTGTCGGAGCAGGAAAAAGACACATTTTGTGAACG

CTTCGGTGTTCGCTTGCTGACGTCTTATGGGATGACGGAAACCATTGTGGGCATTATCGGCGATCGCCCTGGCGATAAAC

GACGCTGGCCGTCGATTGGTCGGGCGGGGTTTTGCTACGACGCGGAGATCCGCGACGATCACAATCGCCCGCTCCCGGCA

GGTGAGATCGGTGAAATCTGTATTAAAGGCGTACCAGGGAAAACCATCTTCAAAGAGTATTTTCTCAACCCGAAAGCCAC

TGCAAAAGTGCTGGAAGCCGATGGTGGGCCTGCATAAA

>15-ecoli-2_F

GTGCCGTTTTTACACTTTCTTTTACAGATCACCAACTCCCTGCCTCGTGTTATTAACCACCCTCTATAATAAAGACTATA

CGGGTGATAATATCATAAATAAGCGAAACCGCTGAGAAAAAGAGTGGTGATGATCAACTATTTCCTGGAGGACTTTGCAT

ATTACTCCTCCTGCGGGCGTGGACCGCGGGACGACGACGTCTACCTGACGAGAATGCCTCCGGTTCACATCGATTGCCAT

GGTACTGCTGCGATGTTGGCGTTTTCTGCCGGAGCCACCTTTCTTTTGGCTGATAAAAACATCCCCCGCGCCTTCTGGGT

GCAGATATATAATTACCTCCCCTCCCATACTTACTGTACGTATACAATGATTTTTATTTTCTTTGTGTGACTCCCGACTG

AAAGATTGTAATCACCATCTGTCTTAATTGATGTTTT

>15-ecoli-3_F

GAATCGTTAACTCGAGACTGATGACGACATCACTGGCGGACGTACTCCGGCCTCCTTCGAGCCGTCCATCGACTACGTGG

TTACCAAAATTCCTCGCTTCAACTTCGAAAAATTCGCCGGTGCTAACGACCGTCTGACCACTCAGATGAAATCGGTTGGC

GAAGTGATGGCGATTGGTCGCACGCAGCAGGAATCCCTGCAAAAAGCGCTGCGCGGCCTGGAAGTCGGTGCGACTGGATT

CGACCCGAAAGTGAGCCTGGATGACCCGGAAGCGTTAACCAAAATCCGTCGCGAACTGAAAGACGCTGGCGCAGAGCGTA

TCTGGTACATCGCCGATGCTTTCCGCGCGGGCCTGTCTGTGGACGGCGTCTTCAACCTGACTAACATTGACCGCTGGTTC

CTGGTACAGATTGAAGAACTGGTGCGTCTGGAAGAGAAAGTGGCGGAAGTGGGCATCACTGGCCTGAACGCTGAATTCCT

GCGCCAGCTGAAACGCAAAGGCTTTGCCGATGCGCGCTTGGCAAAACTGGCGGGCGTACGCGAAGCGGAAATCCGTAAGC

TGCGTGACCAATATGACCTGCACCCGGTCTATAAGCGCGTGGATACCTGTGCGGCAGAGTTCGCCACCGACACCGCTTAC

ATGTACTCCACTTATGAAGAAGAGTGCGAAGGAAAATCCGTCTA

>15-ecoli-4_F

GTTGGTGCGTACGTGCTCACTGACTATCACTACCTGCCACCTTGTGCTATGCACCGCCGCTATCGACTGACGATACGGAG

GATATTCTCTTCACCTCCGGCACCACCTCCCGACCGAAAGGTGTGGTGATTACCCATTACAACCTGCGCTTCGCTGGATA

TTACTCCGCCTGGGAGTGTGCACTGCGTGACGATGACGTCTACCTGACGGTAATGCCTGCGTTTCATATCGATTGCCAGT

GTACTGCGGCGATGGCGGCGTTTTCTGCCGGGGCCACCTTTGTGCTGGTCGAGAAATACAGCGCCCGCGCCTTCTGGGGA

CAGGAGCAGAAGTACCGCGCCACCATTACCGAATGTATTCCGATGATGATTCGTACGTTGATGGTGCAGCCGCCTTCAGC

GAACGATCGGCAACACCGCCTGCGGGAAGTGATGTTTAATCTCAACTTGTCGGAGCAGGAAAAAGATGCGTTTTGTGAAC

GCTTCGGCGTTCGCTTGCTGACGTCTTATGGGATGACGGAAACCATTGTGGGCATTATCGGCGATCGCCCTGGCGATAAA

CGACGCTGGCCGTCGATTGGTCGGGCGGGGTTTTGCTACGAAGCGGAGATCCGCGACGATCACAATCGCCCGCTCCCGGC

AGGTGAGATCGGTGAAATCTGTATTAAAGGCGTACCAGGGAAAACCATCTTCAAAGAGTATTTTCTCAACCCGAAAGCCA

CTGCAAAAGTGCTGGAAGCCGATGGCTGGTCTGTAATAATT

```
                      10        20        30        40        50        60        70        80
                  ....|....|....|....|....|....|....|....|....|....|....|....|....|....|....|....|...
15-ecoli-1_F      ------CTTGGACGGGAGTGCTCACTGACTATCAACAACCTGCCACCTTGTGCTATGCACCGCCGCTATCGACTGACGATACGGAGGA
15-ecoli-2_F      GTGCCGT..TT..ACTTTCTT.T..A..TC.C....TC......T.G.GT.AT..AC....CT.TA..ATA.A-...T......GT..
15-ecoli-3_F      -----GAAGCT.GAT-C..TA.......ATCA.-.................................................C...A
15-ecoli-4_F      -----GT.G.TG..TAC.................-.T...................................................

                     110       120       130       140       150       160       170       180
                  ....|....|....|....|....|....|....|....|....|....|....|....|....|....|....|....|...
15-ecoli-1_F      CTCCGGCACCACCTCCCGACCGAAAGGTGTGGTGATTACCCATTACAACCTGCACTTCGCTGGATATTACTCCGCCTGGGAGTGTGCA
15-ecoli-2_F      TA--A..GAA...G.TGAG--A...A.A.......G.T.A.C..TTT....G.GGA.TT..C..........T....C.G.C...G..
15-ecoli-3_F      ....................................................G..........................C........
15-ecoli-4_F      ....................................................G...................................

                     210       220       230       240       250       260       270       280
                  ....|....|....|....|....|....|....|....|....|....|....|....|....|....|....|....|...
15-ecoli-1_F      GACGTCTACCTGACGATAATGCCTGCGTTTCATATCGATTGCCAGTGTACTGCGGCGATGGCGGCGTTTTCTGCCGGGGCCACCTTTG
15-ecoli-2_F      ................G.......C..G....C...........TG.......T......TT..............A..........C
15-ecoli-3_F      ...............G........................................................................
15-ecoli-4_F      ...............G........................................................................

                     310       320       330       340       350       360       370       380
                  ....|....|....|....|....|....|....|....|....|....|....|....|....|....|....|....|...
15-ecoli-1_F      AATACAGCGCCCGCGCCTTCTGGGGACAGGTACAGAAGTACCGCGCCACCATTACCGAATGTATTCCGATGATGATTCGTACGTTGAT
15-ecoli-2_F      ..A...T.C...............TG...A..T.T..T....T.C..T..CA...TT.C....CGTAT.CA......TT..TT..CT.
15-ecoli-3_F      ...............................G...........................................C............
15-ecoli-4_F      ..............................AG........................................................

                     410       420       430       440       450       460       470       480
                  ....|....|....|....|....|....|....|....|....|....|....|....|....|....|....|....|...
15-ecoli-1_F      TTCAGCGAACGATCGGCAACACCGCCTGCGGGAAGTGATGTTTAATCTCAACTTGTCGGAGCAGGAAAAAGACACATTTTGTGAACGC
15-ecoli-2_F      GA.--T...A...T.TA.T....AT...TCTT..T........T--------------------------------------------
15-ecoli-3_F      ...........................................T...........................TG.G.............
15-ecoli-4_F      .......................................................................TG.G.............

                     510       520       530       540       550       560       570       580
                  ....|....|....|....|....|....|....|....|....|....|....|....|....|....|....|....|...
15-ecoli-1_F      TTGCTGACGTCTTATGGGATGACGGAAACCATTGTGGGCATTATCGGCGATCGCCCTGGCGATAAACGACGCTGGCCGTCGATTGGTC
15-ecoli-2_F      ----------------------------------------------------------------------------------------
15-ecoli-3_F      ........................................................................................
15-ecoli-4_F      ........................................................................................

                     610       620       630       640       650       660       670       680
                  ....|....|....|....|....|....|....|....|....|....|....|....|....|....|....|....|...
15-ecoli-1_F      GCTACGACGCGGAGATCCGCGACGATCACAATCGCCCGCTCCCGGCAGGTGAGATCGGTGAAATCTGTATTAAAGGCGTACCAGGGAA
15-ecoli-2_F      ----------------------------------------------------------------------------------------
15-ecoli-3_F      .......A................................................................................
15-ecoli-4_F      .......A................................................................................

                     710       720       730       740       750       760
                  ....|....|....|....|....|....|....|....|....|....|....|....|....|..
15-ecoli-1_F      AGAGTATTTTCTCAACCCGAAAGCCACTGCAAAAGTGCTGGAAGCCGATGG-TGGGCCTGCATAAA-
15-ecoli-2_F      -------------------------------------------------------------------
15-ecoli-3_F      ..............................G....................C.............--
15-ecoli-4_F      ...................................................C...T.TGTA....TT
```

16-ecoli-marker, Gene Name: Carnitine-CoA transferase (*caiB*)

16-ecoli-F	GGCGGTATACGGGAAGGC
16-ecoli-R	CGTCTCCGGGGGATCTGA

>16-ecoli-1_F

TAGTTCTCACGTTCTGATCAGGTACCACTGAAGGCCTGGGCGATGGTGTTATAGGCCGGAAGATTGGTGTACTCCTCGGT

GCCGTACTGACCAAAACCGGACAGGTGAGCGATAACCAGTTTCGGGTTGTGTTGCCACAGTACTTCATCGGTAATGCCAC

GACGGGCAAAGGCCGGACCTTTACTGGCTTCGATGAAGATATCGGTGGTTTCCATTAATTTCAGAAACGCTTCGCGGCCT

TCATCTTTGAAAATATTTAACGACAGCGCGTGCAAATTGCGGCGGGAGAGTTGCGGGTAGTTCGGTTGAACGCGAATGGT

GTCGGCCCAGGCGACGTTCTCGATCCAGATAACTTCCGCACCCCATTCTGCGAACATTTGCCCGGCGAACGGCCCGGCAA

TTTCGATACCGGAGAAGACAACGCGCAATCCGGCCAACGGCCCGAATTTCGGCATGGGTAGATGATCCATTATTAGCTCC

TGAAAAATTTATGTAGCGCATGACTGCCGGATGCGGCGTAAACGCCTTATCCGGCCTACATTCGTGCTCCCGTAGGCCTG

ATAAGACGCGTCAGCGTCGCATCAGGCAGCGCACGGACTTAGCGGTATTGCTTCAGCACCGCACGACCCAGCGTCAGGAT

CTGCATTTCGTCAGATCCCCCCGGAGACATGAATATCTGGACCTGTGGTGTGGGGTGTTTAAACATTACGCTTAAGTCGT

GCGCTGGCCGAATGCGACGCTTACCGTTTTATCAGCCTACGGGAATCGAAAGGTTGGCCGGATAAGGCTTTTACCCTCAC

CGCGTTCTGCCTCATAAATTTTTCAGGAGTCACAATGGGACTCTCCCTGCCGATTTCGCGCTGTGTGTCTCTGGTCTTTG

TTTCTTCTCTGTTCTATTTTCGCTCTGT

>16-ecoli-2_F

ATGGTTCTCACGTTCTGATCAGGTACCACTGAAGGCCTGGGCGATGGTGTTATAGGCCGGAAGATTGGTGTACTCCTCGG

TGCCGTACTGACCAAAACCGGACAGGTGAGCGATAACCAGTTTCGGGTTGTGTTGCCACAGTACTTCATCGGTAATGCCA

CGACGGGCAAAGGCCGGACCTTTACTGGCTTCGATGAAGATATCGGTGGTTTCCATTAATTTCAGAAACGCTTCGCGGCC

TTCATCTTTGAAAATATTTAACGACAGCGCGTGCAAATTGCGGCGGGAGAGTTGCGGGTAGTTCGGTTGAACGCGAATGG

TGTCGGCCCAGGCGACGTTCTCGATCCAGATAACTTCCGCACCCCATTCTGCGAACATTTGCCCGGCGAACGGCCCGGCA

ATTTCGATACCGGAGAAGACAACGCGCAATCCGGCCAACGGCCCGAATTTCGGCATGGGTAGATGATCCATTATTAGCTC

CTGAAAAATTTATGTAGCGCATGACTGCCGGATGCGGCGTAAACGCCTTATCCGGCCTACATTCGTGCTCCCGTAGGCCT

GATAAGACGCGTCAGCGTCGCATCAGGCAGCGCACGGACTTAGCGGTATTGCTTCAGCACTGCACGACCCAGCGTCAGGA

TCTGCATTTCGTCAGATCCCCCGGAGACATGCAGATCCGGACCTGGGTCTTGCGGTGCTGAAACATTACGCTAATTCGGG

CGCTGCTCGATGCGACGCTTACCCGTCTTAACAGGTCTACGGGAGACGAAATGTTAGCCCGTATAAGCGTTTTACTCGCA

CGCGTCATGCCTCCTTAAATTTTTCAGGACCTAATAGACACCTCCTTTGCCAATTTCTGGCCGTTGCCGAATGTGCTTGT

CTTCGCTCACTATTTGCGCGCTCTCT

>16-ecoli-3_F

AACGTATATCAGCACGTTCTGATCAGGTACCACTGAAGGCCTGGGCGATGGTGTTATAGGCCGGAAGATTGGTGTACTCC

TCGGTGCCGTACTGACCAAAACCGGACAGGTGAGCGATAACCAGTTTCGGGTTGTGCTGCCACAGTACTTCATCGGTAAT

GCCACGACGGGCAAAGGCCGGACCTTTACTGGCTTCGATGAAGATATCGGTGGTTTCCATTAATTTCAGAAACGCTTCGC

GGCCTTCATCTTTGAAAATATTTAACGACAGCGCGTGCAAATTGCGGCGGGAGAGCTGCGGGTAGTTCGGTTGAACGCGA

ATGGTGTCGGCCCAGGCGACGTTCTCGATCCAGATAACTTCCGCACCCCATTCTGCGAACATTTGCCCGGCGAACGGCCC

GGCGATTTCGATACCGGAGAAGACAACGCGCAATCCGGCCAACGGCCCGAATTTCGGCATGGGTAGATGATCCATTATTA

GCTCCTGAAAAATTTATGTAGCGCATGACTGCCGGATGCGGCGTAAACGCTTTATCCGGCCTACATTCGTGCTCCCGTAG

GCCTGATAAGACGCATTAGCGTCGCATCAGGCAGCGCACGGACTTAGCGGTATTGCTTCAGCACCGCGCGACCCAGCGTC

AGGATCTGCATTTCGTCAGATCCCCCGGAGACAAATGCAAATCCGGACCTGGGTGCGCGGTGCTGAAGCATACCGCTTAG

TCGTGCGCTGCCGGATGCGACCTTATGCGTCTTACAGCTACGGAGCACTAAAGTGGCGGATAAGCGTTACCCGCACGCAC

CTGCCTCTAATTTTTTCGGAGCTATATGGATACTCCTGCGAATCTGGCCTGTGCGATGCGCTGTCTTCCTCTCGTATCGA

CGTGCTG

>16-ecoli-4_F

GCATCAGCTCACGTTCTGATCAGGTACCACTGAAGGCCTGGGCGATGGTGTTATAGGCCGGAGAGATTGGTGTACTCCTC

GGTGCCGTACTGACCAAAACCGGACAGGTGAGCGATAACCAGTTTCGGGTTGTGCTGCCACAGTACTTCATCGGTAATGC

CACGACGGGCAAAGGCCGGACCTTTACTGGCTTCGATGAAGATATCGGTGGTTTCCATTAATTTCAGAAACGCTTCGCGG

CCTTCATCTTTGAAAATATTTAACGACAGCGCGTGCAAATTGCGGCGGGAGAGTTGCGGGTAGTTCGGTTGAACGCGAAT

GGTGTCGGCCCAGGCGACGTTCTCGATCCAGATAACTTCCGCCCCCCATTCTGCGAACATTTGCCCGGCAAACGGTCCGG

CGATTTCGATACCGGAGAAGACAACGCGCAATCCGGCCAGCGGCCCGAATTTCGGCATGGGTAGATGATCCATTATTAGC

TCCTGAAAAATTTATGTAGCGCATGACTGCCGGATGCGGCGTAAACGCCTTATCCGGCCGACATTCGTGCTCCCGTAGGC

CTGATAAGACGCGTCAGCGTCGCATCAGGCAGCGCACGGACTTAGCGGTATTGCTTCAGCACCGCGCGACCCAGCGTCAG

GATCTGCATTTCGTCAGATCCCCCGGAGACGATAAATCCGACCTGGGTCCGCCGGTGCTGAACCATACGGCTAATTCGTG

CGCTGCCGATGCGACGCTTAACGTCTTATCAAGGCTACGGAGCACGAAAGGTGGCGGATATGCGTTTATCCAACGCACCT

GCCTCTAAATTTTCGGAACTGACATGGATATCTCCTGCGAAATCTGGCTGCTGACTG

```
                      10        20        30        40        50        60        70        80
              ....|....|....|....|....|....|....|....|....|....|....|....|....|....|....|....|....|...
16-ecoli-1_F  ------TAGTTC-TCACGTTCTGATCAGGTACCACTGAAGGCCTGGGCGATGGTGTTATAGGCCGGA-AGATTGGTGTACTCCTCGGT
16-ecoli-2_F  -----A.G..--.......................................................-....................
16-ecoli-3_F  AACGTA..TCAG--.....................................................-....................
16-ecoli-4_F  -----GC.TCAGC......................................................G....................

                     110       120       130       140       150       160       170       180
              ....|....|....|....|....|....|....|....|....|....|....|....|....|....|....|....|....|...
16-ecoli-1_F  AAAACCGGACAGGTGAGCGATAACCAGTTTCGGGTTGTGTTGCCACAGTACTTCATCGGTAATGCCACGACGGGCAAAGGCCGGACCT
16-ecoli-2_F  ........................................................................................
16-ecoli-3_F  .......................................C................................................
16-ecoli-4_F  .......................................C................................................

                     210       220       230       240       250       260       270       280
              ....|....|....|....|....|....|....|....|....|....|....|....|....|....|....|....|....|...
16-ecoli-1_F  ATGAAGATATCGGTGGTTTCCATTAATTTCAGAAACGCTTCGCGGCCTTCATCTTTGAAAATATTTAACGACAGCGCGTGCAAATTGC
16-ecoli-2_F  ........................................................................................
16-ecoli-3_F  ........................................................................................
16-ecoli-4_F  ........................................................................................

                     310       320       330       340       350       360       370       380
              ....|....|....|....|....|....|....|....|....|....|....|....|....|....|....|....|....|...
16-ecoli-1_F  GCGGGTAGTTCGGTTGAACGCGAATGGTGTCGGCCCAGGCGACGTTCTCGATCCAGATAACTTCCGCACCCCATTCTGCGAACATTTG
16-ecoli-2_F  ........................................................................................
16-ecoli-3_F  ........................................................................................
16-ecoli-4_F  ...................................................................C....................

                     410       420       430       440       450       460       470       480
              ....|....|....|....|....|....|....|....|....|....|....|....|....|....|....|....|....|...
16-ecoli-1_F  CCCGGCAATTTCGATACCGGAGAAGACAACGCGCAATCCGGCCAACGGCCCGAATTTCGGCATGGGTAGATGATCCATTATTAGCTCC
16-ecoli-2_F  ........................................................................................
16-ecoli-3_F  ......G.................................................................................
16-ecoli-4_F  T.....G.....................................G...........................................

                     510       520       530       540       550       560       570       580
              ....|....|....|....|....|....|....|....|....|....|....|....|....|....|....|....|....|...
16-ecoli-1_F  GTAGCGCATGACTGCCGGATGCGGCGTAAACGCCTTATCCGGCCTACATTCGTGCTCCCGTAGGCCTGATAAGACGCGTCAGCGTCGC
16-ecoli-2_F  ........................................................................................
16-ecoli-3_F  .................................T...........................................A.T........
16-ecoli-4_F  ............................................G...........................................

                     610       620       630       640       650       660       670
              ....|....|....|....|....|....|....|....|....|....|....|....|....|....|....|..
16-ecoli-1_F  ACGGACTTAGCGGTATTGCTTCAGCACCGCACGACCCAGCGTCAGGATCTGCATTTCGTCAGATCCCCCCGGAGACA
16-ecoli-2_F  ...........................T.........................................-.......
16-ecoli-3_F  ..............................G......................................-.......
16-ecoli-4_F  ..............................G......................................-......G
```

caiB-4 F : ATA ACC AGT TTC GGG TTG CGC

caiB-4 R : ATC GAA ATC GCC GGA CCG GTT

caiB-3 F :GCA AAT TGC GGC GGG AGG GC

caiB-3 R : CTG CCT GAT GCG ACG TTA AT

20-ecoli primer set, Gene Name: DNA polymerase II (*polB)*

20- ecoli-F	CCCAAGTTGCCCGGTCAT
20- ecoli-R	GCACAGGGCTACGACGTT

>20-ecoli-1_F

GCCCGATAACTCATAAAGGGAGTATTCCCTCCGCCACGGGTTGTAGCTGGCGGGTCAGATAGTGTTCGTAATCCAGTGGT

GAACGTTGGTAGTCCAGCGGCTCCGGGCCGTTGGTGGTCCATACGTACTTAATGGTGCCGCGGTTCTGATATTGCAAGGG

GCGACCGCGCTTTTGGTTTTCTTCATCGGCAAGACGAGCGGCGCGTACATGAGGCGGAACATTACGCTGATATTCGCTCA

GCGGACGGCGAAGGCGTTTACGGTAAACCAGCCGCGCATCCAGTTCACCCGCCATCAGTTTGTCGATGGTTTCGCGGAAG

TACTCCTGATATGGCTCGTTGCGGAAGATACGCAGGTAAAGCTCCTGCTGAAACTGCTGGGCCAGCGGCGTCCAGTCCGT

ACGCACGGTTTCCAGCCCTTTAAACACCATCCGCTGTTTGTCGCCCTCCTGAATCAGCCCGGCATAACGCTTTTTACTGC

CGGTATCGGCTCCGCGAATGGTTGGCATCAGAAAACGGCAGAAATGGGTTTCATACTCCAGTTCCAGTGCGCTGGTCAGC

TGTTGTTTTTGCAGCGTTTCTGCCCACCAGACGTTAACGTGCTGCACCAGTGCACGACCGATTTTCGTCGCTTCTTCTTC

CGAATGTGCGCCTTTCAGCCAGACAAACGTTGAGTCGGTATCGCCGTAGATAACGTCGTAGCCCCTGTGCAAAAAACGTT

AACGTCTGGCGGGGCAAAAACCGTTTTTCTGACTATCCTCTTGACTTTGAATTGGAATTTTGTTTTTTT

>20-ecoli-2_F

GCCCCGTACTTCAATAAAGGTAGTGTTCCCTCCGCCACGGGTTGTAGCTGGCGGGTCAGATAGTGTTCGTAATCCAGTGG

TGAACGTTGGTAGTCCAGCGGCTCCGGGCCGTTGGTGGTCCATACGTACTTAATGGTGCCGCGGTTCTGATATTGCAAGG

GGCGACCGCGCTTTTGGTTTTCTTCATCGGCAAGACGAGCGGCGCGTACATGAGGCGGAACATTACGCTGATATTCGCTC

AGCGGACGGCGAAGGCGTTTACGGTAAACCAGCCGCGCATCCAGTTCACCCGCCATCAGTTTGTCGATGGTTTCGCGGAA

GTACTCCTGATATGGCTCGTTGCGGAAGATACGCAGGTAAAGCTCCTGCTGAAACTGCTGGGCCAGCGGCGTCCAGTCCG

TACGCACGGTTTCCAGCCCTTTAAACACCATCCGCTGTTTGTCGCCCTCCTGAATCAGCCCGGCATAACGCTTTTTACTG

CCGGTATCGGCTCCGCGAATGGTTGGCATCAGAAAACGGCAGAAATGGGTTTCATACTCCAGTTCCAGTGCGCTGGTCAG

CTGTTGTTTTTGCAGCGTTTCTGCCCACCAGACGTTAACGTGCTGCACCAGTGCACGACCGATTTTTCGTCGCTTCTTCT

TCCGAATGTGCGCCTTTCAGCCAGACAAACGTTGAGTCGGTATCGCCGTAGATAACGTCGTAGCCCTTGTGCACAG

>20-ecoli-3_F

GGATACTTCATTAAAGGGAGTATTCCCTCCGCCACGGGTTGTAGCTGGCGGGTCAGATAGTGTTCGTAATCCAGTGGTGA

ACGTTGGTAGACAGGGACCGGGCCGTTGGTGGTCCATACGTACTTAATGGTGCGAGATACTGATATTGCAAGGGGCGACC

GCGCTTTTGATTTTCTTCATCGGCAAGGCGAGCGGCGTGTACATGAGGCGGAACATTACGCTGATATTCGCTCAGCGGAC

GGCGAAGGCGTTTACGGTAAACCAGCCGCGCATCCAGTTCACCCGCCATCAGTTTGTCGATGGTTTGGCGGACGTACTCC

TGATATGGCTCGTTGCGGAAGATACGCAGGTACAGCTCCTGCTGAAACTGCTGGGCCAGCGGCATCCAGTCGGTGCGCAC

GGTTTCCAGCCCTTTAAACTCCATCCGCTGCTTGTCGCCCTCCTGAATAAGCCCGGCATAGCGCTTTTTTATTTTTTTTG

TTTCAACTCCGCGAATGGGTGGTGGGTGGTGAAAACCCCCCCCCCCCCCCTTTCT

>20-ecoli-4_F

GGCATTAACTCATAAAAGGGAGTATTCCCTCCGCCACGGGTTGTAGCTGGCGGGTCAGATAGTGTTCGTAATCCAGTGGT

GAACGTTGGTAGTCCAGCGGCTCCGGGCCGTTGGTGGTCCATACGTACTTAATGGTGCCGCGGTTCTGGTATTGCAAGGG

GCGACCGCGCTTTTGGTTTTCTTCATCGGCAAGGCGAGCGGCGCGTACATGAGGCGGAACATTACGCTGATATTCGCTCA

GCGGACGGCGAAGGCGTTTACGGTAAACCAGCCGCGCATCCAGTTCACCCGCCATCAGTTTGTCGATGGTTTCGCGGACG

TACTCCTGATATGGCTCGTTACGGAAGATACGCAGGTAAAGCTCCTGCTGAAACTGCTGGGCCAGCGGCGTCCAGTCCGT

ACGCACGGTTTCCAGCCCTTTAAACACCATCCGCTGTTTGTCGCCCTCCTGAATCAGCCCGGCATAACGCTTTTTACTGC

CGGTATCGGCTCCGCGAATGGTTGGCATCAGAAAACGGCAGAAATGGGTTTCATACTCCAGTTCTAGTGCGCTGGTCAGC

TGTTGTTTTTGCAGCGTTTCCGCCCACCAGACGTTAACGTGCTGCACCAGTGCACGACCGATTTTCGCCGCTTCTTCTTC

CGAATGTGCGCCTTTCAGCCAGACAAACGTTGAGTCGGTATCGCCGTAGATAAACGTCGGAGCCTCTGTGCAAA

```
                      10        20        30        40        50        60        70        80
                 ....|....|....|....|....|....|....|....|....|....|....|....|....|....|....|....|....|....
20-ecoli-1_F     GCCCGATAACTCA-TAAAGGGAGTATTCCCTCCGCCACGGGTTGTAGCTGGCGGGTCAGATAGTGTTCGTAATCCAGTGGTGAACGTT
20-ecoli-2_F     ....CG..CT...A......T...G...............................................................
20-ecoli-3_F     ---G....CT...T..........................................................................
20-ecoli-4_F     -GG.AT.......TA.........................................................................

                     110       120       130       140       150       160       170       180
                 ....|....|....|....|....|....|....|....|....|....|....|....|....|....|....|....|....|....
20-ecoli-1_F     GCTCCGGGCCGTTGGTGGTCCATACGTACTTAATGGTGCCGCGGTTCTGATATTGCAAGGGGCGACCGCGCTTTTGGTTTTCTTCATC
20-ecoli-2_F     ........................................................................................
20-ecoli-3_F     .GA....................................-.A.A.A..............................A...........
20-ecoli-4_F     .................................................G......................................

                     210       220       230       240       250       260       270       280
                 ....|....|....|....|....|....|....|....|....|....|....|....|....|....|....|....|....|....
20-ecoli-1_F     GGCGCGTACATGAGGCGGAACATTACGCTGATATTCGCTCAGCGGACGGCGAAGGCGTTTACGGTAAACCAGCCGCGCATCCAGTTCA
20-ecoli-2_F     ........................................................................................
20-ecoli-3_F     ....T...................................................................................
20-ecoli-4_F     ........................................................................................

                     310       320       330       340       350       360       370       380
                 ....|....|....|....|....|....|....|....|....|....|....|....|....|....|....|....|....|....
20-ecoli-1_F     TTGTCGATGGTTTCGCGGAAGTACTCCTGATATGGCTCGTTGCGGAAGATACGCAGGTAAAGCTCCTGCTGAAACTGCTGGGCCAGCG
20-ecoli-2_F     ........................................................................................
20-ecoli-3_F     .............G.....C.......................................C............................
20-ecoli-4_F     ...................C.....................A..............................................

                     410       420       430       440       450       460       470       480
                 ....|....|....|....|....|....|....|....|....|....|....|....|....|....|....|....|....|....
20-ecoli-1_F     TACGCACGGTTTCCAGCCCTTTAAACACCATCCGCTGTTTGTCGCCCTCCTGAATCAGCCCGGCATAACGCTTTTTA---CTGCCGGT
20-ecoli-2_F     .............................................................................---........
20-ecoli-3_F     .G........................T..........C.................A...........G........TATTT.TTTT..
20-ecoli-4_F     .............................................................................---........

                     510       520       530       540       550       560       570       580
                 ....|....|....|....|....|....|....|....|....|....|....|....|....|....|....|....|....|....
20-ecoli-1_F     AATGGTTGGCATCAGAAAACGGCAGAAATGGGTTTCATACTCCAGTTCCAGTGCGCTGGTCAGCTGTTGTTTTTGCAGCGTTTCTGCC
20-ecoli-2_F     ........................................................................................
20-ecoli-3_F     .....G...T------GGGT..TGA...CCCCCCC.CCC.C..TT.CT----------------------------------------
20-ecoli-4_F     ................................................T...................................C...

                     610       620       630       640       650       660       670       680
                 ....|....|....|....|....|....|....|....|....|....|....|....|....|....|....|....|....|....
20-ecoli-1_F     ACGTGCTGCACCAGTGCACGACCGATTTT-CGTCGCTTCTTCTTCCGAATGTGCGCCTTTCAGCCAGACAAACGTTGAGTCGGTATCG
20-ecoli-2_F     .............................T..........................................................
20-ecoli-3_F     ----------------------------------------------------------------------------------------
20-ecoli-4_F     .............................-..C.......................................................

                     710       720       730       740       750       760       770       780
                 ....|....|....|....|....|....|....|....|....|....|....|....|....|....|....|....|....|....
20-ecoli-1_F     GTCGTAGCCCCTGTGCAAAAAACGTTAACGTCTGGCGGGGCAAAAACCGTTTTTCTGACTATCCTCTTGACTTTGAATTGGAATTTTG
20-ecoli-2_F     ..........T......C.G--------------------------------------------------------------------
20-ecoli-3_F     ----------------------------------------------------------------------------------------
20-ecoli-4_F     ....G....T.........---------------------------------------------------------------------
```

O-polB-3 F : CAAGGGGCGACCGCGCTTCGA

O-polB-3 R : GCT GGA AAC CGT GCG CCC C

O-polB-4 F : TAATGGTGCCGCGGTTCTGG

O-polB-4 R : CTT TAC CTG CGT ATC TTC AGT

22-ecoli-marker, Gene name: ribulokinase (*araB*)

22-ecoli-F	CCGATTGGCCTGCTTCCA
22- ecoli-R	GCAGCTGTGGTCGGGATT

>22-ecoli-1

CAATATGCTGCTTCCTTTTCCGGGGGCCTGCACGCATAAACGTTAACTGGTTCGTCGTTCAACGCTATAGGGGTGTCGGC

TTCATAAATCGTGGATTAAAGTAAATAATTTTATAGTGCGTTAGGTAGGCGCTTTTTTTATTTTTTGGTCTTATGGCAGT

CATCGCGCCGTTGGAAATCACTACGCTTTTAGGCAGGCCGAGTGTTTGCGCCCACTTTGGGCATAAGGTGCTCACCGGAA

TATAGGCAGTCCATGTTTCAGTGAACAGCGGGGAAGGCAAATGGCATATAAGGATCGGATCCAGCTCATCAAAGAAACTG

GTTGGCGTTCGGCCGCTTTATCTTTCGTGCTATAGCGACTTATGCCTGGCGCTGCTTTTTCCGCGACGAATATCCTGCGG

GCGCGGGGTACCGGAAAGCAGTACTGTCACCCAGCCGCACAGTTGCTCCTCTTTACGGCAGTTTGCTCCTCTTCGCTTTC

CTGGCGAGTCACATGCAGGTTTTGTGTCCATATCCATTCTCTGTAGGAACTACCGCTCACATCGCGTCCAGATATATACT

TATTCCTTTACGTGCCACTTTCAGGTATTGTCTTCTACTGTCTTCCGGCTGCAGCGGGATATTTCCACGTTACGAAAATC

TCTTTAATCGGGATAATTTAAAACTCCGTTGGCGGTTTCTCTACGGTGTATACTTGAATAGCGTCTCTGTTGTTATATCG

ACCCTGGCAACACTGGTAATGGCCGGTGCCGAAGCCATTCTATTCAAAATACTCGTTCTGGGTTGCTTTCT

>22-ecoli-2

CTATTCGTACCCGCTGCATCACCTGACCGCAATACCTTTCACTGCCCGCTCGCCAACGCTCTGTTTGTCGGCAATCAGAA

TGTCGCAGGTGGAAGTACCGATAACTTTTACCAGTGCGTTAGGCTGTGCGCCTGCACCAACTGCGCCCATATGGCAGTCA

AACGCGCCGCCGGAAATCACCACGCTTTCAGGCAGGCCGAGACGCTGCGCCCATTCCGGGCATAAGGTGCCCACCGGAAT

ATCGGCAGTCCAGGTATCAGTGAACAGCGGGGAAGGCAAATGGCGATTGAGGATCGGATCCAGCTCATCAAAGAAACTGG

CTGGCGGCAGGCCGCCCCAGCTTTCGTGCCATAGCGACTTATGCCCGGCGCTGCAACGTCCGCGACGAATATCCTGCGGG

CGCGTGGTACCGGAAAGCAGAGCTGGCACCCAGTCGCACAGCTCAATCCACGATGCGGCAGATTGCGCCACGGCGCTGTC

CTGGCGAGTCACATGCAGGATTTTTGCCCAGAACCATTCGCTGGAATAAATACCGCCAATATAGCGGGAGTAGTCAACAT

TGCCCGGCGCGTGGCACAAACGGGTAATCTCTTCCGCTTCTTCAACCGCAGTGTGGTCTTTCCACAATACGAACATCGCG

TTCGGGTTTTCGGCAAACTCCGGGCGCAGCGCCAGCACGTTACCGTCGGCATCAATCGGTGCGGGCGTCGAGCCGGTACT

GTCAACGCCATCCCCCCCCCAACCGTGCGCCACAATCGGAAGAAATTACCCCTTTTGTGCACACGCGCCTGGTATTGTTT

ACAACCCCGTTTTTTTGGCGTGTTTATATTCCCCGAAAAGGTGTTTTGTGTACATATCTTCCAGATATGGTACTTTCGAT

CATGGGACAAGTGCTAT

>22-ecoli-3

GGAAGGTACCGCTGCATCACCTGACCGCAATACCTTTCACTGCCCGCTCGCCAACGCTCTGTTTGTCGGCAATCAGAATG

TCGCAGGTGGAAGTACCGATAACTTTTACCAGTGCGTTAGGCTGTGCGCCTGCACCAACTGCGCCCATATGGCAGTCAAA

CGCGCCGCCGGAAATCACCACGCTTTCAGGCAGGCCGAGACGCTGCGCCCATTCCGGGCATAAGGTGCCCACCGGAATAT

CGGCAGTCCAGGTATCAGTGAACAGCGGGGAAGGCAAATGGCGATTGAGGATCGGATCCAGCTCATCAAAGAAACTGGCT

GGCGGCAGGCCGCCCCAGCTTTCGTGCCATAGCGACTTATGCCCGGCGCTGCAACGTCCGCGACGAATATCCTGCGGGCG

CGTGGTACCGGAAAGCAGAGCTGGCACCCAGTCGCACAGCTCAATCCACGATGCGGCAGATTGCGCCACGGCGCTGTCCT

GGCGAGTCACATGCAGGATTTTTGCCCAGAACCATTCGCTGGAATAAATACCGCCAATATAGCGGGAGTAGTCAACATTG

CCCGGCGCGTGGCACAAACGGGTAATCTCTTCCGCTTCTTCAACCGCAGTGTGGTCTTTCCACAATACGAACATCGCGTT

CGGGTTTTCGGCAAACTCCGGGCGCAGCGCCAGCACGTTACCGTCGGCATCAATCGGTGCGGGCGTCGAGCCGGTACTGT

CAACGCCAATCCCGCC

>22-ecoli-4

GTAGGTTCCGCTGCATCACCTGACCGCAATACCTTTCACTGCCCGCTCGCCAACGCTCTGTTTGTCGGCAATCAGAATGT

CGCAGGTGGAAGTACCGATAACTTTTACCAGTGCGTTAGGCTGTGCGCCTGCGCCAACTGCGCCCATATGGCAGTCAAAC

GCGCCGCCGGAAATCACCACGCTTTCAGGCAGGCCGAGACGCTGCGCCCATTCCGGGCATAAGGTGCCCACCGGAATATC

GGCAGTCCAGGTGTCAGTGAACAGCGGGGAAGGCAAATGGCGATTGAGGATCGGGTCCAGCTCATCAAAGAAACTGGCTG

GCGGCAAGCCACCCCAGCTTTCGTGCCATAGCGACTTATGCCCGGCGCTGCAACGTCCGCGACGAATATCCTGCGGGCGG

GTGGTACCGGAAAGCAGAGCTGGCACCCAGTCGCACAGCTCAATCCACGATGCGGCAGATTGCGCCACGGCGGTGTCCTG

GCGAGTCACATGCAGGATTTTTGCCCAGAACCATTCGCTGGAATAAATACCGCCAATGTAGCGGGAGTAGTCAACGTTGC

CCGGCGCGTGGCACAAACGGGTAATCTCTTCCGCTTCTTCAACCGCAGTGTGGTCTTTCCACAATACGAACATCGCGTTC

GGGTTTTCGGCAAACTCCGGGCGCAGCGCCAGCACGTTACCGTCGGCATCAATCGGTGCGGGCGTCGAGCCGGTTGTGTC

AACGCCAATCCCGACACACAAGGCTGC

```
                          10        20        30        40        50        60        70        80
                 ....|....|....|....|....|....|....|....|....|....|....|....|....|....|....|....|....|...
22-ecoli-1_F     CAATATGCTGCTTCCTTTTCCGGGGGCCTGCACGCATAAACGTTAACTGGTTCGTCGTTCAACGCTATAGGGGTGTCGGCT-TCATAA
22-ecoli-2_F     ----C.AT.CG.A..CGC.G.ATCA-....AC....AT.C.T..C....CCCGC...C-.......C.--.TT.......AA...G..
22-ecoli-3_F     -----G.AA.G.A..-GC.G.ATCA-....AC....AT.C.T..C....CCCGC...C-.......C.--.TT.......AA...G..
22-ecoli-4_F     ------.TA.G....-GC.G.ATCA-....AC....AT.C.T..C....CCCGC...C-.......C.--.TT.......AA...G..

                         110       120       130       140       150       160       170       180
                 ....|....|....|....|....|....|....|....|....|....|....|....|....|....|....|....|....|...
22-ecoli-1_F     AAGTA--AATAATTTT--ATAGTGCGTTAGGTAG-GCGCTTTTTTTATTTTTTGGTCTTATGGCAGTCAT-CGCGCCGTTGGAAATCA
22-ecoli-2_F     .....CCG....C...TACC...........CT.T....C.GCACC.AC.GC-.CC.A...........AA.......CC........
22-ecoli-3_F     .....CCG....C...TACC...........CT.T....C.GCACC.AC.GC-.CC.A...........AA.......CC........
22-ecoli-4_F     .....CCG....C...TACC...........CT.T....C.GCGCC.AC.GC-.CC.A...........AA.......CC........

                         210       220       230       240       250       260       270       280
                 ....|....|....|....|....|....|....|....|....|....|....|....|....|....|....|....|....|...
22-ecoli-1_F     GCAGGCCGAGTGTTTGCGCCCACTTTGGGCATAAGGTGCTCACCGGAATATAGGCAGTCCATGTTTCAGTGAACAGCGGGGAAGGCAA
22-ecoli-2_F     ..........ACGC........T.CC.............C...........C.........G..A.......................
22-ecoli-3_F     ..........ACGC........T.CC.............C...........C.........G..A.......................
22-ecoli-4_F     ..........ACGC........T.CC.............C...........C.........G..G.......................

                         310       320       330       340       350       360       370       380
                 ....|....|....|....|....|....|....|....|....|....|....|....|....|....|....|....|....|...
22-ecoli-1_F     GATCGGATCCAGCTCATCAAAGAAACTGGTTGGCGTTCGGCCGCTTTATCTTTCGTGCTATAGCGACTTATGCCTGGCGCTGCTTTTT
22-ecoli-2_F     .............................C.....GCA......CCC.G.........C...............C........AACG.
22-ecoli-3_F     .............................C.....GCA......CCC.G.........C...............C........AACG.
22-ecoli-4_F     ......G......................C.....GCAA...A.CCC.G.........C...............C........AACG.

                         410       420       430       440       450       460       470       480
                 ....|....|....|....|....|....|....|....|....|....|....|....|....|....|....|....|....|...
22-ecoli-1_F     TCCTGCGGGCGCGGGGTACCGGAAAGCAGTACTGTCACCCAGCCGCACAG-TTGCTCCTCTTTACGGCAGTTTGCTCCTCTTCGCTTT
22-ecoli-2_F     .............T...............AG...G.......T.......C.CAA...A.GA.G......A....G..A.GG....G.
22-ecoli-3_F     .............T...............AG...G.......T.......C.CAA...A.GA.G......A....G..A.GG....G.
22-ecoli-4_F     ...........G.T...............AG...G.......T.......C.CAA...A.GA.G......A....G..A.GG..G.G.

                         510       520       530       540       550       560       570       580
                 ....|....|....|....|....|....|....|....|....|....|....|....|....|....|....|....|....|...
22-ecoli-1_F     CATGCAGGTTTTGTGTCCATATCCATTCTCTGTAGGAACTACCGCTCACATCGCGTCCAGATATATACTTATTCCTTTACGTGCCACT
22-ecoli-2_F     ........A...T..C...G.A......G...G.AT..A......CA.T..A...GG-..TAG.CA..A.TGC..GGCG....G...A
22-ecoli-3_F     ........A...T..C...G.A......G...G.AT..A......CA.T..A...GG-..TAG.CA..A.TGC..GGCG....G...A
22-ecoli-4_F     ........A...T..C...G.A......G...G.AT..A......CA.TG.A...GG-..TAG.CA..G.TGC..GGCG....G...A

                         610       620       630       640       650       660       670       680
                 ....|....|....|....|....|....|....|....|....|....|....|....|....|....|....|....|....|...
22-ecoli-1_F     CTTCTACTGTCTTCCGGCTGCAGCGGGATATTTCCACGTTACGAAAATCTCTTTAATCGGGATAATTTAAAACTCCGTTGGCGGTTTC
22-ecoli-2_F     ....CG..--TC.T.AA.C....T.T.G.C.......AA......C...G.G..---....T.TTCGGC........GGC..A.CGC.
22-ecoli-3_F     ....CG..--TC.T.AA.C....T.T.G.C.......AA......C...G.G..---....T.TTCGGC........GGC..A.CGC.
22-ecoli-4_F     ....CG..--TC.T.AA.C....T.T.G.C.......AA......C...G.G..---....T.TTCGGC........GGC..A.CGC.

                         710       720       730       740       750
                 ....|....|....|....|....|....|....|....|....|....|..
22-ecoli-1_F     ACTTGAATAG-CGTCTCTGTTGTTATATCGACCCTGGCAACACTGGTAATGG
22-ecoli-2_F     T.GGC.TC.AT..GTG.G.GC..CGAGC..GTA...T....G.CATCCCCCC
22-ecoli-3_F     T.GGC.TC.AT..GTG.G.GC..CGAGC..GTA...T....G.CAA.CCC.C
22-ecoli-4_F     T.GGC.TC.AT..GTG.G.GC..CGAGC..GTTG..T....G.CAA.CCC.A
```

26-ecoli marker, Gene Name: 3-isopropylmalate dehydratase subunit (*leuD*)

26-ecoli-F	CAGTGGCGGCAGGAGTAC
26-ecoli-R	CCCTGGGCATTGACCGAC

>26-ecoli-1

GTAAATTTCGGGGTCGGCAGGTCGCAGTTTCAGCAGCTCTTTAATTTCTGCTTCTTTCGCACCGTCGAACACCGGCGTTG

CGATTGGCATACCTTTGCGCAGGTTTTCAGCCAGACGCATAACTTCTTCATCGCTGAAGGTACTCAGGTCAACTTTCTGA

CGAACGTCAGCGCCCAGATCGTACGCACGCTGGATGAATTCACGCAGTTTCGCGACTTCCTGCTGCTGTTTCAGCATGGC

GTTGATCTTGTCGCCGATACCTTTCGCAGCCATACCCAAGTGGGTTTCGAGGATCTGACCGATGTTCATACGAGACGGTA

CGCCCAGCGGGTTCAGTACGATGTCTACCGGCGTACCGTTTTCATCGTAAGGCATATCTTCGATCGGGTTGATCTTAGAA

ATTACACCCTTGTTACCGTGACGACCTGCCATCTTGTCACCAGGCTGGATACGGCGTTTAACCGCCAGATATACCTTAAC

AATCTTCAGCACGCCCGGTGCCAGATCGTCGCCCTGGGTGATTTTGCGGCGTTTCGCTTCGAGTTTCTTCTCGAACTCGT

GTTTCAGTTCGTCATACTGCTCAGCCAGCTGTTCCAGCTGATTTTGTTTCTCTTCGTCGGTCATTTGCTTAAAAAATGAC

CTGCTAATACTGCAAACTCTACAGATTTTCAGGAGATCAGCAATCGTTCGGGGCAATGCCCAGGGCCCCCCTGGTTA

>26-ecoli-2

TAAATATCAGAGTCGGCAGGTCGCCAGTTTCAGCAGCTCTTTAATTTCTGCTTCTTTCGCACCGTCGAACACCGGCGTTG

CGATTGGCATACCTTTGCGCAGGTTTTCAGCCAGACGCATAACTTCTTCATCGCTGAAGGTACTCAGGTCAACTTTCTGA

CGAACGTCAGCGCCCAGATCGTACGCACGCTGGATGAATTCACGCAGTTTCGCGACTTCCTGCTGCTGTTTCAGCATGGC

GTTGATCTTGTCGCCGATACCTTTCGCAGCCATACCCAAGTGGGTTTCGAGGATCTGACCGATGTTCATACGAGACGGTA

CGCCCAGCGGGTTCAGTACGATGTCTACCGGCGTACCGTTTTCATCGTAAGGCATATCTTCGATCGGGTTGATCTTAGAA

ATTACACCCTTGTTACCGTGACGACCTGCCATCTTGTCACCAGGCTGGATACGGCGTTTAACCGCCAGATATACCTTAAC

AATCTTCAGCACGCCCGGTGCCAGATCGTCGCCCTGGGTGATTTTGCGGCGTTTCGCTTCGAGTTTCTTCTCGAACTCGT

GTTTCAGTTCGTCATACTGCTCAGCCAGCTGTTCCAGCTGATTTTGTTTCTCTTCGTCGGTCATGGGCCCAGGGAGAGTT

TCTATTTTTGCCAGCTTACCCGATCTCATCATATTCTCATGGGCGGTCAATGCCGAATGTTTTGTGCCGCTTTTA

>26-ecoli-3

TACAAGTTACATTTCTTTCATAGCGTATGGCGTTGATGACGCTGCAACTTTTTAACGCTGTATTTATCGGCATTGTTGCG

GGTATTGGGATGCTATGGTTTCAGGATTTAATGCCTGGAAGAGCGGGGGCAGCTACCACCTTATTTACTAACAGTATTTC

TACCGGGGTAATTCTGGCTGGCGTTATTCAGGGAGCAATTGCACAAAGTTGGGGGCACTTTGCTGTCTACTGGGTAATTG

CGGTTATTTCTGTTGTCGCATTATTTTTAACCGCAAAGGTTAAAGACGTTTGATGACGTGGACGATAGCGGAAAGCCCGG

TCATTTGACCGGGCAAGGGGATTAATTCATAAACGCAGGTTGTTTTGCTTCATAAGCGGCAATGGCGTCGTCGTGCTGCA

AGGTAAGCCCAATACTGTCCAGACCGTTCATCATGCAGTGGCGGCGGAAGGCATCGATGGTAAAGCGATAGGTTTTCTCT

CCCGCTTTCACCTCTTGCGCTTCCAGATCCACGTCGAAATGGATCCCCGGATTGGCTTTCACCAGCGCAAACAGTTCGTC

CACTTCTGCATCGCTTAATTTCACCGGCAGCAGCTGGTTGTTAAAGCTATTGCCGTAGAAGATGTCAGCAAAACTCGGCG

CAATCACCACTTTAAAACCGTAGTCGGTCATGGCCCCAGGGA

>26-ecoli-4

GTGCCCTCGCAGTAGCGATTTCTCGCGCTGTTCCTCTTCGTTGATACCCAGAATACCAAACACCGTCTGCTGCATATCAC

CATTATGGATACGTACTGAACCACCGCCCACTTCGTAACCATTGATGACCATATCGTAAGCGTTCGCCACCGCATTTTCC

GGTGCAGCTTTCAGCTCTGCAGCCGTCATGTCTTTCGGTGAGGTGAACGGATGGTGCATTGCCGTCAGGCCGCCTTCACC

GTCGTCTTCAAACATCGGGAAGTCGATAACCCACAGCGGTGCCCATTTGCTTTCGTCGGTCAATGCCCGGGGTTTACCCA

CTTTCAGGCGCAGTGCGCCCATCGCGTCGGCAACAATTTTCTTGTTGTCGGCACCGAAGAAAATCATATCGCCATCTTGC

GCGGCAGTACGATCCAGGATGGCTTCGATGATTTCAGCATTAAGGAACTTCGCTACCGGGCTGTTGATACCTTCCAGACC

TTTCGCGCGTTCGTTAACTTTGATGTAAGCCAGACCTTTCGCGCCGTAGATTTTAACGAAGTTACCGTATTCGTCGATCT

GCTTACGGGTCAGCGATGCGCCACCCGGAACACGCAGAGCGGCAACGCGACCTTTCGGATCGTTCGCCGGACCTGCAAAT

ACTGCAAACTCAACAGACTTCAGCAGATCAGCAACGTCGGTCATTTGCCCCAGGAGA

```
                   10        20        30        40        50        60        70        80
           ....|....|....|....|....|....|....|....|....|....|....|....|....|....|....|....|....|...
26-ecoli-1_F  TCGGCAGGTCGC-AGTTTCAGCAGCTCTTTAATTTCTGCTTCTTTCGCACCGTCGAACACCGGC---GTTGCGATTGGCATACCTTTG
26-ecoli-2_F  ...........C....................................................---.....................
26-ecoli-3_F  --TA..A..TA.ATT.C.TTCATAGCG.A.GGCG.TGATGA.GC.GCA..TT.TT...G.T.TATTTA.C.GC....TTGCGGG.A.T
26-ecoli-4_F  C.CT.GCAGTAGCGA....TCGC...G..CCTC...GTTGATACC.AG.ATAC.A.......T.T--.C...A--.AT..CCAT.A..

                  110       120       130       140       150       160       170       180
           ....|....|....|....|....|....|....|....|....|....|....|....|....|....|....|....|....|...
26-ecoli-1_F  GCCAGACGCATAACTTCTTCATCGCTGAAGGTACTCAGGTCAACTTTCTGACGAACGTCAGCGCCCAGATCGTACGCACGCTGGATGA
26-ecoli-2_F  ........................................................................................
26-ecoli-3_F  TTTCAGGATT...TGC..GG.AGAGC.GG..C.GCT.--C..C...AT.T..T...AGT.TTT.T---.C..GGGTA.TT....C..G
26-ecoli-4_F  A...C-...-CC.....G.A.C.AT...T.ACCA.ATC..A.G.G...--G.C.C..CATTTT..GGTGCA.CTTT..GCTCT.CA.C

                  210       220       230       240       250       260       270       280
           ....|....|....|....|....|....|....|....|....|....|....|....|....|....|....|....|....|...
26-ecoli-1_F  TCGCGACTTCCTGCTGCTGTTTCAGCATGGCGTTGATCTTGTCGCCGATACCTTTCGCAGCCATACCCAAGTGGGTTTCGAGGATCTG
26-ecoli-2_F  ........................................................................................
26-ecoli-3_F  -A..A.T.G.ACAAA.T..GGGGCA.T.T..TG.CTA..G.GTAATTGCGGT.A.TT.T.TTG.CG.ATTA.TTT.AA.CGCA.AGGT
26-ecoli-4_F  CG.T..GG.GA-A.G.A..G.---....T..CG.C.GGCC.C.TT.ACCGT.G.CTT..AA...CGGG....C.A.AA.CCAC.G.G.

                  310       320       330       340       350       360       370       380
           ....|....|....|....|....|....|....|....|....|....|....|....|....|....|....|....|....|...
26-ecoli-1_F  ATACGAGACGGTACGCCCAGCGGGTTCAGTACGATGTCTACCGGCGTACCGTTTTCATCGTAAGGCATATCTTCGATCGGGTTGATCT
26-ecoli-2_F  ........................................................................................
26-ecoli-3_F  ..GACGTGGACG.TAG.GGAAA.CCCGGTC.TTTGAC.GGG.AAG.GGATTAA....AAACGCAGG.TGT..T.C.----.CATAAG
26-ecoli-4_F  T.T..TCGGTCA.T....G.-..T..ACCC..TT.CAGGCG.A.T.CG..CA.CG.G...GC.ACA..T.TC.T.T.GTC.GCACCGA

                  410       420       430       440       450       460       470       480
           ....|....|....|....|....|....|....|....|....|....|....|....|....|....|....|....|....|...
26-ecoli-1_F  CCTTGTTAC-CGTGACGACCTGCCATCTTGTCACCAGGCTGGATACGGCGTTTAACCGCCAGATATACC---TTAACAATCTT-CAGC
26-ecoli-2_F  .........-...........................................................---...........-....
26-ecoli-3_F  .G.C..GCTG.AA.GTA.---...CAA.AC.GT....A.C.--.T.AT.A.GC.GTG..GGCGG.AGG.--A.CG.TGG.AAAG.GAT
26-ecoli-4_F  .GCCA.CTTG..C.G.AGTAC.ATCCAGGA.GG.TTC.A..AT.T.A..A..A.GGAA.TTCGC-....GGGC.GTTG..ACC-TTC.

                  510       520       530       540       550       560       570       580
           ....|....|....|....|....|....|....|....|....|....|....|....|....|....|....|....|....|...
26-ecoli-1_F  AGATCGTCGCCCTGGGTGATTTTGCGGCGTTTCGCTTCGAGTTTCTTCTCGAACT---CGTGTTT-CAGTTCGTCATACTGCTCAGCC
26-ecoli-2_F  .......................................................---.......-......................
26-ecoli-3_F  CCGCTT..A..TCTT.C.C..CCAGA----.C.A.G....AA.GGA..C.CGGA.---T.GC...-..CCAGCG..A..A.T..GT..
26-ecoli-4_F  C.T....TAA.T.T.A..TAAGCCA.A.C......GC..TAGA.T..AA....G.TAC...A..CGTC.A..TG.T...G.G.....G

                  610       620       630       640       650       660       670       680
           ....|....|....|....|....|....|....|....|....|....|....|....|....|....|....|....|....|...
26-ecoli-1_F  GCTGATTTTGTTTCTCTTCGTCGGTCATTTGCTTAAAAAAT-GACCTGCTAATACTGCAAACTCTACAGATTT--TCAGGAGATCAGC
26-ecoli-2_F  ............................GG..CC.GGG.---..GT.T...T.TT...C.G..TAC.C...C.CA...TATTC...TG
26-ecoli-3_F  CT.A....CACCGGCAGCA.CT...TG..AAAGCT.TTGCCGT.GAA.A.GTC.GCA-......GG.GC.A.C-AC..CTTT.A-.A.
26-ecoli-4_F  .AAC.CGCA.AGCGG.AA..CGACCTT.CG.A.CGTTCGCCG......A.............A.....C..CAG...ATC.G..A.

                  710       720
           ....|....|....|....|....|
26-ecoli-1_F  C-AATGCCCAGGGCCCCCCTGGTTA
26-ecoli-2_F  .G...TTTT.T...G.TT.TA---
26-ecoli-3_F  T--GGC.......A-----------
26-ecoli-4_F  .-----......AGA----------
```
